# Phenolic Contents and Compositions in Skins of Red Wine Grape Cultivars among Various Genetic Backgrounds and Originations

**DOI:** 10.3390/ijms13033492

**Published:** 2012-03-14

**Authors:** Lei Zhu, Yali Zhang, Jiang Lu

**Affiliations:** 1 College of Food Science and Nutritional Engineering, China Agricultural University, Beijing 100083, China; E-Mails: Zhulei610@gmail.com (L.Z.); olivia.yl.zhang@gmail.com (Y.Z.); 2 Center for Viticulture and Small Fruit Research, Florida Agricultural and Mechanical University, Tallahassee, FL 32317, USA

**Keywords:** *Vitis*, skin, phenolic content, phenolic compositon, principal component analysis

## Abstract

In order to analyze and compare the phenolic characteristics of red wine grapes with diverse genetic backgrounds, skin phenolics among 21 different cultivars belonging to *Vitis vinifera* L., East Asian and North American *Vitis* species and hybrids, as well as 2 varieties of muscadine grapes were estimated by HPLC-MS/MS. There were 45 anthocyanins, 28 flavonols, 8 flavan-3-ols, 9 cinnamic acids, 5 benzoic acids, 5 ellagic acids and 2 stilbenes detected in all the samples. Total contents of each phenolic type varied significantly among the different grape cultivars investigated. There was also a large variability in the phenolic compositions of different grape groups. The differences in anthocyanin composition were obvious between *V. vinifera* and non-*V. vinifera* grapes and also between the grapes originating from Eurasia and North America. Quercetin-3-glucuronide and quercetin-3-glucoside were marker flavonol compounds for *Euvitis* grape skins. Flavan-3-ol monomers were dominant in the skins of muscadine and non-*V. amurensis* East Asian grapes, whereas polymers were more common in *V. vinifera* and North American grapes. The muscadine grapes were very rich in flavonols, flavan-3-ols and ellagic acids. Via principal component analysis, these grape cultivars were clustered into three groups according to their characteristic phenolic content and composition.

## 1. Introduction

The grape (*Vitis* L.), which has a long history of cultivation and utilization, is one of the most important commercial fruit crops worldwide. There are more than 70 grape species and a large number of grape cultivars growing all over the world [1]. *Vitis* L. is divided into two subgenera: *Euvitis* Planch. and *Muscadinia* Planch. Based on geographical distributions, there are three groups of *Euvitis* species, including *V. vinifera*, East Asian and North American *Vitis* species. *Muscadinia* Planch., which refers mainly to *V. rotundifolia* Michx., is originated in the southeastern United States. This grape is also called muscadines and is genetically distinct from *Euvitis* species [2].

With more than 30 grape species being reported, China is the most important original center of East Asian *Vitis* species [3]. *Vitis vinifera*, the predominant *Vitis* species distributed and cultivated worldwide today, is believed to have been introduced to China more than 2000 years ago. At present, wine grape varieties cultivated in China mostly belong to *V. vinifera* plus a small percentage of Oriental species and hybrids. Recently, more American grapes and French hybrids for wine were introduced to China and received a great deal of attention due to their strong disease and pest resistance and stress tolerance [1].

Phytochemicals in grapes are mostly phenolic compounds. According to their molecular structure, the phenolic compounds are divided into four classes: one phenolic ring (cinnamic acids and benzoic acids), two phenolic rings (stilbenes), three rings (anthocyanins, flavonols and flavan-3-ols) and complex ring (ellagic acids) [4]. Anthocyanins, flavonols and flavan-3-ols, which have the nuclear molecular structure of C_6_-C_3_-C_6_, are also called flavonoid compounds. The remaining compounds are termed non-flavonoids. Grape skins contain abundant, widely varied phenolics. These phenolics play an important role in the sensory properties and nutrition of berries and wines. Many studies demonstrated that these phenolics could reduce the incidence of serious chronic diseases such as cancer and cardiovascular diseases, due to their antioxidant abilities [4–6].

Liquid chromatography-tandem mass spectrometry (HPLC-MS) technology has become a popular tool for the efficient identification of phenolic compounds. As a result, a lasrge amount of phenolic compounds have been identified and quantified in the grape berries and wines from *V. vinifera* [7–9], muscadines [10–12] and some hybrids [13,14]. However, few reports have reported phenolic content and coposition in different grape species/cultivars originated or cultivated in China, especially non-anthocyanin compounds. Thus the purpose of the present study was to perform an extensive analysis of phenolic compositions in the skins of a selected group of red wine grape cultivars with very diverse genetic backgrounds (Table 1). This information will be very useful for evaluating the winemaking and nutritional potential of these different grapes.

## 2. Results and Discussion

### 2.1. Anthocyanin Profiles

Total anthocyanins (TAs) of all the samples ranged from 1065.63 to 16840.99 μg Malvidin-3-glucoside (MGE) /g dry weight (DM) (Table 2). There were large differences in the TAs of differernt grapes, even among cultivars / species from the same original region. The skin of American hybrid “St. Croix” had the largest amount of TA (16840.99 μg MGE/g DM), while the smallest amounts of TA were found in *V.labrusca* “Catawba” and “Niagara Rosada” (1457.04 and 1065.63 μg MGE/g DM), which were native to North America. Among the East Asian grapes, *V. amurensis* “Zuoshanyi”, “Zuoshaner” and *V. ficifolia* “Sangye”, and a *V. amurensis* × *V. vinifera* hybrid “Zuohongyi”, produced high amounts of anthocyanins in their skins (more than 10000 μg MGE/g DM). Meanwhile, *V. dividii* “Black Pearl”, *V.xunyangensis* “Mi” and *V.quinquangularis* “Mao” had low amounts of TAs (1000–2000 μg MGE/g DM).

A total of 45 anthocyanin compounds were identified from all the samples, including monoglucoside, diglucoside and their acylated derivatives of six anthocyanidins: delphinidin (dp), cyanidin (cy), petunidin (pt), pelargonidin (pg), peonidin (pn) and malvidin (mv) (Table 3). The mv-derivatives were the dominant anthocyanin type in the grape skins of *V. vinifera* (77.8%) and East Asian species (53.55%), in which mv-3-glucoside (glc) and mv-3,5-diglc were, respectively, the most anthocyanin. The chief anthocynin types in most North American grapes were cy- (43.21%) and dp- derivatives (32.20%) due to their high levels of cy-3-glc and dp-3-glc. In the case of *V. rotundifolia* grapes, only the diglucosides of the six anthocyanidins were found, where dp-3,5-glc was the most abundant anthocyanin in “Alachua” (45.16%) and cy-3,5-glc was the most abundant in “Noble” (36.17%). Moreover, pt-3,5-glc (21.44% on average) was relatively abundant in the skins of both cultivars. In the skins of the hybrids, anthocyanins were primarily represented by mv-, dp- and pt-derivatives, but the proportions were different in Euro-Asian hybrids (24.20%, 43.34% and 19.59% of TAs, respectively) and Euro-American hybrids (47.56%, 24.54% and 21.24% of TAs, respectively). Small amounts of pg-3,5-diglucosides were detected only in *V. rotundifolia* “Alachua” and “Noble”, and pg-3-glucoside was detected in *V.labrusca* “Niagara Rosada” and *V.aestivalis* “Black Spanish”.

According to the numbers of B-ring substituents of anthocyanidins [14], the anthocyanins detected could be divided into three groups: 4′-substituents (pg-derivatives), 3′,4′-substituents (cy- and pn-derivatives) and 3′,4′,5′-substituents (dp-, pt- and mv-derivatives). In general, 3′,4′,5′-substituted anthocyanins were dominant in the skins of *V. vinifera* grapes, East Asian grapes, and Euro-Asian and Euro-American hybrids, accounting for 87.57% of TA on everage. Muscadine and North American grapes were different, where 3′,4′- and 3′,4′,5′-substituted anthocyanins accounted for 52.93% and 45.89% of TAs, respectively, and 4′-substituted anthocyanins were minor components.

Anthocyanins can also be classified into non-methylated (pg-, dp- and cy-derivatives) and methylated ones (pt-, pn- and mv-derivatives). The proportions of methylated anthocyanins in the TAs of the skins decreased in the order: *V. vinifera* grapes (94.22%) > East Asian grapes (77.30%) > Euro-American hybrids (72.49%) > Euro-Asian hybrids (49.10%) > muscadine grapes (42.86%) > North American grapes (24.23%).

With regard to monoglucoside and diglucosides anthocyanins, there was a distinct separation among the different species. The *V. vinifera* grapes only contained the monoglucoside anthocyanins, but *V. rotundifolia* grapes only had diglucosides anthocyanins. Among the East Asian species and *V. amurensis* × *V. vinifera* hybrids, diglucosides anthocyanins were dominant in most grapes (accounting for 86.81% of TAs on average) except *V.quinquangularis* “Mao” and the hybrids “Zuohongyi” and “Hasang”. While monoglucoside anthocyanins were the main anthocyanins in North American grapes and Euro-American hybrids (accounting for 74.76% of TAs on average), except *V.aestivalis* “Black Spanish” and the hybrid “St. Croix”.

Among acylated anthocyanins, the *p*-coumaroyl derivatives were the most abundant ones with a high propotion (21.61% of TAs on average) in most samples with the exception of *V. amurensis* and *V. rotundifolia* grapes*,* in which no acylated anthocyanins were detected. The percentage of acetyl derivatives was high in the two *V. vinifera* grapes, but very low in non-*V. amurensis* East Asian grapes, North American grapes, and Euro-Asian and Euro-American hybrids.

Colored anthocyanins play an important role in the quality of red grapes and wines, which make the anthocyanin profile a major index for classifying grape cultivars and wine. In anthocyanin biosynthesis, there are three branches from naringenin: 4′-substituted, 3′,4′-substituted and 3′,4′,5′-substituted anthocyanins. The latter two have an absolute advantage among grape anthocyanins, but their proportion of TAs largely depends on the expression level of flavonoid 3′-hydroxylase and flavonoid 3′,5′-hydroxylase. Meanwhile the expression levels of methyltransferase, glucosyltransferase and acetyltransferase directly influence the degree of methylation, glucosylation and acylation [15]. As a result, the grapes with different genotypes displayed diverse anthocyanin compositions. Liang *et al.* (2008) concluded malvidin derivatives were the most abundant anthocyanins in the majority of germplasms, and monoglucoside derivatives were in *V. vinifera* and both mono- and di-glucoside derivatives in other *Vitis* germplasms. Their data were based on the analysis of the anthocyanin composition in the skins of 110 *Euvitis* grape cultivars [16]. For muscadine grapes, 3,5-diglucosides of delphinidin, cyanidin and petunidin accounted for approximately 90% of TAs [17].

In the present study, we found some interesting trends worth investigating in further research in the molecular mechanism differences of anthocyanin biosynthesis in red wine grapes with various genetic backgrounds and originations. i) Methylated and 3′,4′,5′-substituted anthocyanins were dominant in the skins of *V. vinifera* and East Asian grapes native to Eurasia, the proportion of which was significantly higher than in North American and muscadine grapes native to American. ii) Mono-glucosides were the only anthocyanin type in European grape skins and dominant in North American grape skins, while di-glucoside anthocyanins were the only type in muscadines and dominant in East Asian grapes. iii) The proportion of acetyl anthocyanins was significantly higher in *V. vinifera* grapes than in non-*V. vinifera* grapes.

### 2.2. Flavonol and Dihydroflavonols Profiles

Flavonols convert from dihydroflavonols through the formation of a double bond between C-2 and C-3 in the dihydroflavonol molecules under the function of flavonol synthase [18]. So the compounds belonging to flavonols and dihydroflavonols can be considered as one kind of phenolics [19,20] which are the most abundant non-anthocyanin components found in the grape skins. Total flavonols and dihydroflavonols (TFOs), ranging from 67.08 to 1892.53 μg quercetin equivalence (QE) /g DW, varied widely among the skins of the different grapes investigated (Table 2). *V. rotundifolia* “Noble” (1892.53 μg QE/g DW) and “Alacha” (1856.99 μg QE/g DW) had almost 2-fold higher TFO contents than the highest bunch grapes which were *V. ficifolia* “Sangye” (1358.17 μg QE/g DW), *V. labrusca* “Concord” (200.41 μg QE/g DW), and “Niagara Rosada” (1024.86 μg QE/g DW). Most *V. amurensis* cultivars (“Shuanghong”, “Shuangyou” “Zuoshanyi” and “Zuoshaner”) and the French hybrid “Marechal Foch” had less than 200 μg QE/g DW.

Among the 28 detected compounds (Table 4), there were six flavonol aglycones (quercetin, kaempferol, myricetin, isorhamnetin, syringetin, laricitrin) and two dihydroflavonol aglycones (dihydroquercetin and dihydrokaempferol). Quercetin derivatives were dominant in all the samples, accounting for 68.84% of TFOs on everage. Quercetin-3-*O*-glucuronide and quercetin-3-*O*-glucoside, accounting for 22.49% and 19.01% of TFOs, respectively, were the most common quercetin derivatives in the skins of all cultivars except muscadines, in which quercetin-3-*O*-rhamnoside (34.81%) and quercetin (26.97%) were the main flavonols. Most *V. amurensis* cultivars and hybrids were rich in quercetin, ranging from 20%–50% of TFOs, while non-*V. amurensis* East Asian species were dominated by quercetin-3-*O*-rhamnoside (42.44% of TFOs on average).

Kaempferol derivatives were the second most important flavonols in the skins of North American grapes and Euro-American hybrids, accounting for 24.04% and 10.19% of TFOs, respectively. However, myricetin derivatives were the second most abundant ones in East Asian grapes, Euro-Asian hybrids and muscadine grapes, accounting for 10.39%, 15.54% and 13.34% of TFOs, respectively. The proportions of other flavonol aglycone derivatives were low in most samples except the two *V. vinifera* grapes, in which isorhamnetin, dihydroquercetin, syringetin and kaempferol derivatives accounted for 18.49%, 14.69%, 14.36% and 12.54% of TFOs, respectively.

Flavonols are related to bitterness, and act as copigments of anthocyanins in the wines [21]. Many previous studies investigated flavonol composition in *V. vinifera* grape skins [22–24]. Our research was the first to show that quercetin-3-glucuronide and quercetin-3-glucoside were the characteristic flavonols in the skins of *Euvitis* red grapes. These compounds were not detected in muscadine samples. The large amount of diverse flavonols and dihydroflavonols was an outstanding characteristic of flavonol profiles for the skins of muscadine grapes, which might enhance their antioxidant capacity and make their taste different.

### 2.3. Flavan-3-ol Profiles

Flavan-3-ols mainly contribute to the structure of wines. Flavanol polymers (condensed tannin) play a particularly important role in the astringency of wines. They may react with anthocyanins through an intermolecular copigmentation process, leading to the definition and stability of color in red wines [25]. There were significant differences in total content and chemical compositions of flavan-3-ols (TFAs) among various grape cultivars (Table 2). Among American cultivars and Euro-American hybrids, *V. labrusca* “Niagara Rosada” had the highest TFA content in all the tested samples (1243.67 μg CE/g DW), while *V. labrusca* “Catawba”, *V.aestivalis* “Black Spanish”, and the French hybrid “Marechal Foch” had less than 20 μg catechin equivalence (CE) /g DW. The two muscadines also possessed high TFAs (451.48μg CE/g DW for “Noble” and 350.62 μg CE/g DW for “Alachua”). A relatively low TFA contents was found in most East Asian cultivars, and none were detected in most *V. amurensis* cultivars and its hybrids except “Hasang” and “Changbaijiu” (562.73 and 381.80 μg CE/g DW).

There were 8 flavan-3-ol compounds detected in all, including 4 monomers (gallocatechin, epigallocatechin, catechin, epicatechin), 3 dimers and 1 trimer (Table 4). In the skins of the *V. vinifera* grapes, North American grapes and Euro-American hybrids, the flavan-3-ols were mainly comprised of polymers, accounting for 70%–100% of TFAs, *e*xcept in the French hybrid “Marechal Foch”. However, monomers were the chief flavan-3-ol type in the muscadines and non-*V. amurensis* East Asian species. The most abundant monomers were found in *V. rotundifolia* “Noble” and “Alachua” (338.20 and 265.63 μg CE/g DW, respectively). Among all the flavan-3-ols, the procyanidin dimer was the most important compound in the non-muscadine grapes. The high levels of TFA in the skins of “Niagara Rosada”, “Hasang” and “Changbaijiu” were correlated to their extremely high contents of procyanidin dimmer levels. For monomers, catechin and epicatechin were the most common, but their levels were still low in most non-muscadine grapes.

### 2.4. Non-Flavonoid Phenolic Profiles

The total cinnamic acids (TCA) contents in the tested samples ranged from trace amounts to 230 μg caffeic acid equivalence (CAE)/g DW (Table 2). The three cultivars with the highest levels of TCA were *V. xunyangensis* “Mi”, the *V. amurensis* × *V. vinifera* hybrid “Zuoyouhong” and *V. dividii* “Black pearl” (229.37, 219.42 and 216.19 μg CAE/g DW, respectively). In addition, *V. quinquangularis* “Mao”, *V. labrusca* “Fredonia” and *V. amurensis* “Changbaijiu” also had a high TCA content (174.13, 154.96 and 109.73 μg CAE/g DW, respectively). However, only trace cinnamic acids were detected in *V. vinifera* “Merlot” and “Cabernet Sauvignon”. Nine different cinnamic acids were found in the skins among the cultivars investigated, with chlorgenic acid, hexose ester of ferulic acid, and fertaric acid were more common than others. Fertaric acid was the most abundant cinnamic acids in the East Asia group, accounting for 49.87% of TCAs (Table 5).

Five benzoic acids were also identified, and hexose ester of vanillic acid was the most common, followed by hexose ester of chlorgenic acid (Table 5). In general, low benzoic acids were detected in all the cultivars (Table 2). For example, the highest total benzoic acid content (TBC) was only 27.85 μg gallic acid equivalence (GAE)/g DW, which was found in *V. ficifolia* “Sangye”, whereas the lowest content was found in *V. labrusca* “Catawba” (0.58 μg GAE/g DW).

Ellagic acids were uniquely found in muscadine grapes. There were 647.68 and 525.16 μg ellagic acid equivalence (EAE)/g DW of total ellagic acids (TEAs) in “Noble” and “Alachua”, respectively (Table 2). Hydrolyzable tannins including hexahydroxydiphenoic (HHDP)-glucose, HHDP-galloylglucose and 2 ellagitannins were dominant. Among them, ellagitannins were the most abundant, accounting for 69.78% (“Noble”) and 75.56% (“Alachua”) of TEAs (Table 5).

Two stilbene compounds, trans-resveratrol and its glucoside (*trans*-pecied), were identified in all the tested grape berry skins (Table 5). The highest level of total stilbenes (TS) level was observed in *V. Vinifera* “Merlot” skins (167.06 μg RE/g DW), followed by *V. ficifolia* “Sangye” and *V. rotundifolia* “Noble” (75.76 and 71.67 μg RE/g DW, respectively). There were trace or no stilbenes found in American grape cultivars and Euro-American hybrids, and also in most *V. amurensis* cultivars and hybrids, except “Shuangyou”, “Zuohongyi” and “Hasang” (Table 2).

### 2.5. Principle Component Analysis

Twenty-two evaluation parameters in all the cultivars investigated (4′-substituent, 3′,4′-substituent, 3′,4′,5′-substituent, methylated, non-methylated, monoglucoside, diglucosides, acetyl, caffeoyl, coumaroyl and total anthocyanins; quercetin, myricetin, kaempferol derivatives and total dihydroflavonols and flavonols; monomeric, polymeric and total flavon-3-ols; total cinnamic acids, total benzoic acids, total ellagic acids and total stilbenes) were subjected to Principle Component Analysis (PCA) in order to separate these grapes according to their skin phenolic characteristics. The first three principal components (PCs) possessed relatively high percentages of total variance. PC1 described 27.94% of the variance, which had high contributions from 4′-substituted anthocyanins, quercetin derivatives, myricetin derivatives, total flavonols, and monomeric and total flavon-3-ols, as well as total ellagic acids. PC2 described 25.32% of the variance, and correlated with 3′,4′-substituted, 3′,4′,5′-substituted, methylated, non-methylated, diglucosides, total anthocyanins and total benzoic acids. PC3 accounted for 12.50% of the variance and was mostly described by kaempferol derivatives, and polymeric and total flavon-3-ols.

**Figure 1:**
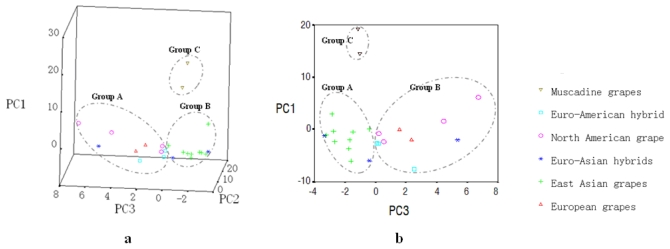
Distributions of the 23 grape cultivars in the three-dimensional space of principle component (PC)1, PC2 and PC3 (**a**) and the two-dimensional space of PC1 and PC3 (**b**).

Figure 1 shows the distribution of the 23 grapes in the three-dimensional space of PC1, PC2 and PC3 and the two-dimensional space of PC1 and PC3. The score plots of these samples revealed three distinct groups. Group A, located in the positive axis of PC3, included all the *V. Vinifera*, North American grape cultivars, Euro-American hybrids, and the *V. amurensis* × *V. vinifera* hybrid “Hasang”. This group was linked by their relatively high contents of kaempferol derivatives or polymeric or total flavon-3-ols in the skins. Group B, in the negative PC3 axis, was comprised of all the East Asian grapes and Euro-Asian hybrids except “Hasang”. This group was matched through no or low content of kaempferol derivatives or polymeric or total flavon-3-ols in their skins. The two mascadine grapes comprised group C, located in the significantly higher position of the PC1 axis. This group was characterized by the presence of pg-3,5-diglucosides and ellagic acids, and by high contents of monomeric flavan-3-ols and flavonols.

## 3. Experimental Section

### 3.1. Materials

Berries of the five *V. amurensis* grape cultivars, three *V. amurensis* × *V. vinifera* hybrids, four American cultivars and three Euro-American hybrids were collected from the experimental vineyard of the grape germplasm repository of China Agricultural University in Beijing. The two *V. vinifera* grape cultivars were from the nearby vineyard of Ji county in Tianjing. The berries of the other four East Asian species were collected from the experimental vineyard of the grape germplasm repository of Henan Academy of Agricultural Science in Zhengzhou. The berries of the two muscadine grape cultivars were collected from the experimental vineyard of Guangxi Academy of Agricultural Science in Nanning (Table 1).

All the grape berries were harvested at technological ripeness upon ripening in 2009, determined based on the former years ripening dates and as judged from seed color change to dark brown without senescence of berry tissue. Two 100-berry batches were sampled from at least 50 cluster selections at similar positions of 6 whole vine selections. Each group of berries was considered as one replication resulting in two replications for every grape cultivar or hybrid.

The fresh samples were placed in refrigerated bags and taken to the laboratory. Then the skins were separated manually from the berries and immediately freeze-dried (LGJ-12, Songyuan Huaxing Corporation, Beijing, China). Dried specimens were ground thoroughly with a stainless-steel grinder (FW-135, Taister Corporation, Tianjin, China), and stored in vacuum-packaged polyethylene pouches at −20 °C for subsequent analysis.

### 3.2. Chemicals and Standards

The standards, caffeic acid, gallic aid, resveratrol, malvidin-3-*O*-glucoside, quercetin, catechin and ellagic acid, were all obtained from Sigma-Aldrich (St. Louis, MO). The HPLC grade reagents, methanol, formic acid, acetic acid and acetonitrile, were purchased from Fisher Scientific Co. (Fairlawn, NJ, USA). Folin-Ciocalteu phenol reagent (2 N) and 2,4,6-tripyridyl-*s*-triazine (TPTZ) (≥99%) were purchased from Sigma-Aldrich (St. Louis, MO, USA). 6-Hydroxy-2,5,7,8-tetramethylchro-man-2-carboxylic acid (Trolox) (≥99%) was purchased from Alexis (Axxora, Switzerland). All other analytical grade chemicals and reagents were purchased from Lanyi Co. (Beijing, China).

### 3.3. Extraction of Phenolic Compounds

A protocol developed in our lab was used for the total phenol extraction and anthocyanin analysis [26]. Briefly, 20 mL of a solution methanol/water/acetic acid (70:29:1, v/v/v) was added into 50 mL centrifuge tube that contained 0.5 g of grape skin powder. The tubes were placed in a shaker (SHZ-88A, Taicang Experiment Equipment Factory, Jiangsu, China) with 300 rpm for 2 h at 25 °C in a dark environment. After that, the turbid extraction liquids were separated by a centrifuge (Beckman Coulter Ltd, Palo Alto, CA, USA) to collect the supernatants. The residues were repeated the above procedure for two times. The samples were then stored at −20 °C in dark until analysis.

For the analysis of non-anthocyanin phenolics including phenolic acids, stilbenes, flavonols, flavan-3-ols and ellagic acids, the extraction was performed according to Jin, He, Bi, Cui and Duan (2009) [19]. Briefly, two gram skin powder was weighed into 100 mL conical flask with 4 mL distilled water and 20 mL ethyl acetate, shaking with 300 rpm for 30 min in darkness. After centrifuged, the residues were re-extracted four times. All collected supernatants were evaporate to dryness using a rotary evaporator (RE-52A, Yarong biochemistry instrument factory, Shanghai, China) under 30 °C and re-dissolved with methanol to the unified volume of 2 mL. The final solutions were filtered through 0.22 μm Nylon membrane filters for HPLC-MS analysis.

### 3.4. Analysis of Phenolic Compounds by HPLC-MS/MS

Anthocyanin analysis were performed on an Agilent 1100 series LC-MSD trap VL equipped with a G1379A Degasser, a G1311A QuatPump, a G1313A ALS, a G1316A Column, a G1315B DAD and a Kromasil-C18 column (250 × 4 mm, 6.5 μm). The mobile phase was: aqueous 2% formic acid as solvent A, and acetonitrile containing 2% formic acid as solvent B. The gradient profile was from 6% to 10% B for 4 min, from 10% to 25% B for 8 min, isocratic 25% B for 1 min, from 25% to 40% for 7 min, from 40% to 60% for 15 min, from 60% to 100% for 5 min, and from 100% to 6% for 5 min. The flow rate was 1.0 mL·min^−1^ and the column temperature was set at 50 °C. The injection volume was 30 μL and the detection wavelength was 525 nm. MS analyses was used Electrospray ionisation (ESI), positive ion model, 35 psi nebulizer pressure, 10 mL min-1 dry gas flow rate, 350 °C dry gas temperature, and 100–1000 *m*/*z* scan range [19].

Analysis of non-anthocyanins were performed on an Agilent 1200 series equipped with a G1322A Degasser, a G1312B Bin pump, a G1367C HiP-ALS, a G1316B TCC, a G1314C VWD and a ZORBAX SB-C18 column (3 × 50 mm, 1.8 μm). The mobile phase was: water solution with 1% acetic acid as solvent A, and acetonitrile solution with 1% acetic acid as solvent B. The gradient profile was from 5% to 8% solvent B for 10 min, from 8% to 10% B for 8 min, from 10% to 15% B for 22 min, from 15% to 20% B for 10 min, from 20% to 30% B for 3 min, from 30% to 50% B for 5 min, from 50% to 100% B for 4 min, and isocratic 100% B for 4 min. The flow rate was 1.0 mL·min^−1^ and the column temperature was set at 25 °C. The injection volume was 2 μL and the detection wavelength was 280 nm. MS analyses were used as follows: Electrospray ionisation (ESI) interface, negative ion model, 35 psi nebulizer pressure, 10 mL·min-1 dry gas flow rate, 325 °C dry gas temperature, and 100–1000 *m*/*z* scan range [20].

Each group of phenolic compounds was quantified using its representative compound as standard. Anthocyanins, flavonol, flavan-3-ols, cinnamic acids, benzoic acid, stilbenes and ellagic acids were respectively expressed as micrograms of Malvidin-3-glucoside (MGE), quercetin equivalence (QE), catechin equivalence (CE), caffeic acid equivalence (CAE), gallic acid equivalence (GAE), resveratrol equivalence (RE) and ellagic acid equivalence (EAE)/g of dry matter (DM).

### 3.5. Statistical Analysis

All analyses were expressed as means ± standard deviations (S.D.) of duplicate. All data were subjected to one-way analysis of variance (ANOVA) using SPSS 16.0 (SPSS Inc.) at the 95% confidence level. The principal component analysis (PCA) was performed to investigate skin phenolic-based species relationships and correlate phenolic compositions to species/cultivars.

## 4. Conclusions

Skin phenolic compositions varied widely among grape cultivars with different genetic backgrounds and originations. Monoglucoside and diglucoside anthocyanins were only detected in *V. vinifera* grapes and *V. rotundifolia* grapes, respectively, while both existed in other species and hybrids. In the grapes originating from Eurasia, 3′,4′,5′-substituted and methylated anthocyanins were dominant, while the proportions of total anthocyanins were relatively low in grapes originating from North America. In addition, acetyl anthocyanins were more abundant in *V. vinifera* grapes than other species grapes. Furthermore, quercetin-3-glucuronide and quercetin-3-glucoside were common in the skins of *Euvitis* species but were not detected in muscadine grapes. The muscadine grape skins possessed more non-anthocyanin phenolics than other grapes due to their richness in flavonols, flavan-3-ol monomers and ellagic acids. Flavan-3-ols were generally sparse in the skins of the East Asian grapes and hybrids. According to PCA, all the samples could be divided into three groups: *V. rotundifolia* grapes, East Asian grapes and hybrids, and other grapes.

## Supplementary Information



## Figures and Tables

**Table 1:** Grape cultivars belonging to different groups and species used in this study.

Grape groups and species	cultivars
European grapes	
*V. vinifera*	Cabernet Sauvignon (CS), Merlot (ML)

East Asian grapes	
*V. amurensis*	Changbaijiu (CB), Shuanghong (SH), Shuangyou (SY), Zuoshanyi (ZS-1), Zuoshaner (ZS-2)
*V. dividii*	Black Pearl (BP)
*V.quinquangularis*	Mao (MA)
*V.xunyangensis*	Mi (MI)
*V. ficifolia*	Sangye (SN)

Euro-Asian hybrids	
*V. amurensis*, *V. vinifera*	Hasang (HS), Zuohongyi (ZH), Zuoyouhong (ZY)

North American grapes	
*V.aestivalis*	Black Spanish (BS)
*V.labrusca*	Catawba (CT), Concord (CC), Niagara Rosada (NR)

Euro-American hybrids	
*V. riparia*, *V. rupestris*, *V. vinifera*	Marechal Foch (MF)
*V. rupestris*, *V. lincecumii*, *V. vinifera*	Chambourcin (CH), St. Croix (SC)

Muscadine grapes	
*V. rotundifolia*	Alachua (AL), Noble (NB)

**Table 2:** Total contents of each phenolic group in the skins of the grape cultivars.

Cultivars	Total anthocyanins (TA)	Total flavonols (TFO)	Total flavan-3-ols (TFA)	Total cinnamic acids (TCA)	Total benzoic acid (TBA)	Total ellagic acid (TEA)	Total stilbenes (TS)
European grapes							
CS	4163.28 ± 151.49 ^e,f^	611.75 ± 39.84 ^g,h,I^	60.89 ± 0.97 ^a^	1.33 ± 0.00 ^a^	8.64 ± 0.30 ^b,c,d,e,f^	nd	26.83 ± 0.86 ^c^
ML	3158.11 ± 323.03 ^d^	315.83 ± 41.98 ^b,c,d^	209.84 ± 47.39 ^a,b,c,d^	tr	10.17 ± 1.95 ^b,c,d,e,f,g^	nd	167.06 ± 7.21 ^e^
East Asian grapes							
CB	4656.70 ± 72.26 ^g^	438.97 ± 13.61 ^e,f,g,h^	381.80 ± 12.38 ^c,d,e^	109.73 ± 2.51 ^g^	9.49 ± 0.74 ^b,c,d,e,f,g^	nd	tr
SH	6595.13 ± 103.62 ^i^	199.3 ± 1.75 ^a,b,c,d^	nd	70.53 ± 2.93 ^e,f,g^	15.30 ± 0.01 ^f,g,h,i,j^	nd	nd
SY	4479.19 ± 125.29 ^f,g^	67.08 ± 3.03 ^a^	nd	78.80 ± 2.57 ^f,g^	7.92 ± 0.03 ^b,c,d,e^	nd	3.79 ± 0.45 ^a^
ZS-1	10036.96 ± 113.72 ^m^	192.27 ± 2.26 ^a,b,c,d^	nd	38.08 ± 0.46 ^a,b,c,d,e^	6.53 ± 1.38 ^a,b,c^	nd	tr
ZS-2	14370.94 ± 16.04 ^o^	113.29 ± 0.36 ^a,b^	nd	61.90 ± 0.97 ^d,e,f^	11.54 ± 0.93 ^c,d,e,f,g^	nd	tr
BP	1607.43 ± 62.31 ^b,c^	392.36 ± 0.88 ^d,e,f,g^	16.46 ± 1.14 ^a^	216.19 ± 27.17 ^i^	12.86 ± 1.93 ^c,d,e,f,g^	nd	2.83 ± 0.37 ^a^
MA	1192.31 ± 85.32 ^a,b^	385.74 ± 11.02 ^d,e,f^	16.08 ± 7.05 ^a^	174.13 ± 1.29 ^h^	7.09 ± 0.11 ^a,b,c^	nd	22.37 ± 0.80 ^b,c^
MI	1801.37 ± 64.34 ^c^	326.26 ± 18.68 ^b,c,d,e^	14.30 ± 4.83 ^a^	229.37 ± 7.66 ^i^	21.70 ± 0.60 ^j,k,l^	nd	2.17 ± 0.14 ^a^
SN	11580.58 ± 127.70 ^n^	1358.17 ± 123.03 ^k^	69.96 ± 2.62 ^a^	22.68 ± 1.07 ^a,b,c,d^	27.85 ± 1.28 ^l^	nd	75.76 ± 0.72 ^d^
Euro-Asian hybrids							
HS	8029.36 ± 184.13 ^k^	613.97 ± 48.46 ^h,i^	562.73 ± 308.23 ^e^	55.04 ± 0.49 ^c,d,e,f^	7.32 ± 3.25 ^a,b,c,d^	nd	27.31 ± 5.15 ^c^
ZH	13901.93 ± 47.90 ^o^	359.46 ± 15.11 ^c,d,e^	nd	49.83 ± 1.18 ^b,c,d,e,f^	14.19 ± 0.54 ^d,e,f,g,h^	nd	3.64 ± 0.05 ^a^
ZY	4421.08 ± 78.47 ^f,g^	241.00 ± 13.68 ^a,b,c,d,e^	nd	219.42 ± 38.59 ^I^	15.90 ± 0.35 ^g,h,i,j^	nd	nd
North American grapes							
BS	8842.82 ± 18.65 ^l^	626.81 ± 25.98 ^h,i^	6.45 ± 0.64 ^a^	49.43 ± 2.86 ^b,c,d,e,f^	14.25 ± 3.41 ^e,f,g,h,i^	nd	tr
CT	1457.04 ± 13.47 ^a,b,c^	277.09 ± 13.61 ^a,b,c,d,e^	9.66 ± 1.64 ^a^	28.72 ± 2.88 ^a,b,c,d^	0.58 ± 0.33 ^a^	nd	nd
CC	5792.99 ± 258.15 ^h^	1200.41 ± 34.15 ^j,k^	181.26 ± 3.30 ^a,b,c^	77.80 ± 0.13 ^f,g^	12.73 ± 0.14 ^c,d,e,f,g^	nd	nd
NR	1065.63 ± 12.84 ^a^	1024.86 ± 47.31 ^j^	1243.67 ± 28.54 ^f^	20.43 ± 1.01 ^a,b,c^	3.43 ± 0.92 ^a,b^	nd	nd
Euro-American hybrids							
MF	2949.30 ± 87.68 ^d^	138.70 ± 12.40 ^a,b,c^	15.85 ± 0.34 ^a^	13.29 ± 0.14 ^a,b^	6.08 ± 0.01 ^a,b,c^	nd	tr
CH	8450.64 ± 20.74 ^k,l^	612.25 ± 6.95 ^g,h,i^	180.89 ± 15.60 ^a,b,c^	51.22 ± 3.59 ^b,c,d,e,f^	14.39 ± 0.13 ^e,f,g,h,i^	nd	tr
SC	16840.99 ± 60.59 ^p^	376.81 ± 10.77 ^d,e,f^	55.80 ± 14.96 ^a^	33.08 ± 6.43 ^a,b,c,d,e^	14.42 ± 4.35 ^e,f,g,h,i^	nd	nd
Muscadine grapes							
AL	2920.11 ± 25.80 ^d^	1856.99 ± 202.38 ^l^	350.62 ± 36.67 ^b,c,d,e^	57.98 ± 6.81 ^c,d,e,f^	14.03 ± 0.91 ^d,e,f,g,h^	525.16 ± 123.42^a^	12.15 ± 4.17 ^a,b^
NB	5548.91 ± 6.36 ^h^	1892.53 ± 53.60 ^l^	451.48 ± 48.98 ^d,e^	24.89 ± 2.53 ^a,b,c,d^	21.09 ± 3.56 ^i,j,k,l^	647.68 ± 148.81 ^b^	71.67 ± 12.51 ^d^

Values are means of duplicate determination ± S.D. nd means not detected. tr means trace. Each abbreviation represents a cultivar, see Table 1. Different letters in each column are significantly different at 0.05 level from ANOVA.

**Table 3:** Retention times, electrospray ionisation (ESI)-MS/MS *m*/*z* values (molecular ion (MS); product ions (MS^2^)), average contents and range in parentheses of anthocyanin compounds in the skins of different grape groups (μg MGE /g FW).

Compounds	Rt (min)	MS; MS^2^ (*m*/*z*)	Eu-grapes	As-grapes	Eu-As hybrids	Am-grapes	Eu-Am hybrids	Mu-grapes
Dp-3,5-diglc	3.45	627;465,303	nd	896.43 (0–2378.51)	562.78 (106.45–839.73)	214.45 (0–644.79)	440.01 (89.48–1038.96)	926.30 (533.73–1318.86)
Cy-3,5-diglc	3.54	611;449,287	nd	415.83 (0–1143.16)	263.13 (14.46–452.11)	166.45 (83.65–272.07)	206.92 (29.23–529.53)	1285.33 (563.84–2006.81)
Pt-3,5-diglc	3.80	641;479,317	nd	858.62 (33.51–2734.42)	315.21 (33.73–474.79)	156.89 (0–499.54)	596.19 (35.66–1426.36)	895.45 (653.75–1137.16)
Dp-3-glc	4.43	465;303	123.39 (123.18–123.60)	606.35 (0–2634.50)	2968.24 (505.87–5679.48)	600.33 (141.58–1137.04)	1156.07 (343.65–2383.92)	nd
Pg-3,5-diglc	4.63	595;433,271	nd	nd	nd	nd	nd	97.18 (31.59–162.77)
Pn-3,5-diglc	5.33	625;463, 301	nd	416.21 (37.87–897.11)	254.24 (41.84–429.82)	195.07 (0–568.77)	262.11 (36.16–498.54)	678.16 (190.00–1166.33)
Mv-3,5-diglc	5.73	655;493, 331	nd	2371.40 (133.01–4663.20)	1366.12 (53.16–2547.98)	367.52 (0–1277.69)	1940.85 (268.32–2950.67)	352.08 (162.06–542.11)
Cy-3-glc	6.25	449;287	47.79 (47.21–48.37)	nd	197.68 (0–593.03)	508.30 (40.07–741.42)	nd	nd
Pt-3-glc	7.33	479;317	122.43 (116.90–127.97)	194.89 (0–748.27)	1266.11 (96.60–2153.69)	136.83 (6.12–252.17)	800.43 (404.02–1105.79)	nd
Pg-3-glc	8.70	433;271	nd	nd	nd	14.24 (0–47.45)	nd	nd
Cy-3-acglc-5-glc	8.96	653;611,449,287	nd	0 (0-tr)	nd	nd	2.36 (0–7.08)	nd
Pt-3-acglc-5-glc	9.28	683;641,479,317	nd	nd	nd	15.60 (0–62.39)	46.71 (0–140.13)	nd
Pn-3-glc	10.02	463;301	241.23 (218.63–263.83)	51.48 (0–272.50)	122.68 (29.55–268.92)	71.01 (12.70–134.85)	79.60 (39.56–106.07)	nd
Mv-3-glc	11.01	493;331	1794.77 (1436.27–2153.27)	202.71 (27.38–967.11)	655.32 (95.39–1458.67)	78.12 (0–201.12)	1094.53 (488.69–1634.41)	nd
Dp-3-cfglc-5-glc	11.48	789;627,465,303	nd	nd	nd	19.95 (0–79.81)	nd	nd
Dp-3-*cis*-cmglc-5-glc	12.07	773;611,465,303	nd	nd	nd	30.37 (0–91.26)	nd	nd
Pn-3-acglc-5-glc	12.30	667;625,463,301	nd	nd	nd	nd	nd	nd
Dp-3-acglc	12.50	507;465,303	nd	nd	nd	18.29 (0–73.15)	85.36 (34.04–169.32)	nd
Mv-3-acglc-5-glc	12.90	697;655,493,331	nd	4.35 (0–24.29)	nd	15.17 (0–60.69)	53.20 (27.98–91.20)	nd
Dp-3-*trans*-cmglc-5-glc	14.55	773;611,465,303	nd	4.38 (0–16.54)	75.36 (0–226.08)	405.99 (0–1304.32)	605.56 (18.45–1666.21)	nd
Cy-3-acglc	14.73	491;449,287	nd	nd	nd	10.54 (0–42.15)	nd	nd
Dp-3-cfglc	14.96	627;465,303	nd	0.67 (0–6.07)	21.86 (0–65.57)	nd	nd	nd
Pt-3*-cis*-cmglc-5-glc	15.49	787;625,479,317	nd	0.81 (0–7.30)	4.97 (0–14.90)	15.98 (0–55.27)	8.87 (0–26.62)	nd
Pt-3-acglc	16.22	521;317	nd	0 (1-tr)	21.86 (0–65.58)	22.68 (0–45.40)	52.70 (35.50–77.05)	nd
Dp-3-*cis*-cmglc	17.22	611;303	nd	1.10 (0–9.86)	nd	nd	nd	nd
Cy-3-cmglc-5-glc	17.58	757;595,449,287	nd	nd	20.35 (0–61.05)	166.05 (0–336.70)	101.12 (0–289.80)	nd
Pt-3*-trans*-cmglc-5-glc	17.78	787;625,479,317	nd	6.95 (0–40.05)	59.08 (0–177.25)	206.07 (0–691.57)	404.10 (46.92–973.12)	nd
Mv-3-cfglc-5-glc	18.22	817;655,493,331	nd	10.70 (0–79.97)	nd	nd	nd	nd
Pt-3-cfglc	19.01	641;479,317	nd	1.24 (0–11.20)	nd	nd	16.90 (10.43–24.90)	nd
Pn-3*-cis*-cmglc-5-glc	19.23	771;609,463,301	nd	0 (0-tr)	nd	8.45 (0–21.84)	nd	nd
Mv-3*-cis*-cmglc-5-glc	19.56	801;639,493,331	nd	6.87 (0–24.03)	nd	nd	16.08 (0–48.24)	nd
Dp-3-*trans*-cmglc	20.11	611;303	nd	8.99 (0–67.26)	390.23 (0–1170.68)	319.51 (7.03–798.26)	575.88 (38.37–1456.98)	nd
Pn-3-acglc	20.33	505;301	55.85 (51.03–60.67)	nd	nd	23.02 (0–92.07)	3.55 (0–10.65)	nd
Mv-3-acglc	20.87	535;331	511.05 (414.43–607.68)	2.06 (0–18.51)	10.02 (0–30.07)	12.44 (0–33.86)	52.88 (15.20–114.01)	nd
Cy-3-*cis*-cmglc	21.03	595;287	nd	nd	nd	5.34 (0–13.49)	nd	nd
Pn-3*-trans*-cmglc-5-glc	21.35	771;609,463,301	nd	nd	nd	104.79 (0–328.97)	nd	nd
Mv-3*-trans*-cmglc-5-glc	21.69	801;639,493,331	nd	139.26 (0–583.23)	18.80 (0–56.41)	123.78 (0–391.45)	348.75 (45.62–702.93)	nd
Pt-3-*cis*-cmglc	22.02	625;317	nd	0.93 (0–8.36)	nd	nd	nd	nd
Mv-3-cfglc	23.41	655;331	38.05 (26.26–49.85)	1.44 (0–12.96)	47.97 (0–143.90)	158.01 (36.06–454.79)	65.05 (10.99–157.78)	nd
Cy-3-*trans*-cmglc	24.67	595;287	nd	1.82 (0–16.38)	2.58 (0–7.74)	nd	19.96 (0–39.02)	nd
Pt-*trans*-cmglc	25.84	625;317	72.60 (69.43–75.78)	9.50 (0–75.70)	114.79 (0–344.38)	56.37 (0–132.89)	191.91 (28.41–346.48)	nd
Pn-3-*cis*-cmglc	26.50	609;301	nd	0 (0-tr)	nd	nd	nd	nd
Mv-3-*cis*-cmglc	27.60	639;331	nd	2.46 (0–22.12)	nd	nd	4.37 (0–13.12)	nd
Pn-3-*trans*-cmglc	29.23	609;301	98.17 (93.57–102.76)	25.26 (0–219.00)	3.66 (0–10.97)	16.27 (0–37.45)	10.95 (7.98–13.14)	nd
Mv-3-*trans-*cmglc	29.66	639;331	555.35 (544.50–566.21)	15.12 (0–70.23)	21.08 (0–63.23)	25.74 (0–55.25)	170.65 (49.41–313.01)	nd

nd means not detected. tr means trace. Abbreviations: Eu, European; As, East Asian; Am, North Amrican; Mu, Muscadine; Dp, delphinidin; Cy, cyanidin; Pt, petunidin; Pg, pelargonidin; Pn, peonidin; Mv, malvidin; diglc, diglucosides; glc, glucoside; acglc, (6-acetyl)-glucoside; cfglc, (6-caffeoyl)-glucoside; cmglc, (6-coumaroyl)-glucoside. The contents of the anthocyanin compounds in the skins of each wine grape cultivars are shown in the supplementary file.

**Table 4:** Retention times, ESI-MS/MS *m*/*z* values (molecular ion (MS); product ions (MS^2^)), average contents and range in parentheses of dihydroflavonol, flavonol and flavan-3-ol compounds in the skins of different grape groups.

Compounds	Rt (min)	MS; MS^2^ (*m*/*z*)	Eu-grapes	As-grapes	Eu-As hybrids	Am-grapes	Eu-Am hybrids	Mu-grapes
*Dihydroflavonols and flavonols (μg QE/g DW)*								
Dk-3-glc	5.00	449;287	nd	nd	nd	nd	nd	66.68 (0–133.35)
K-3-hex	5.98	447;285	nd	nd	nd	32.47 (0–53.11)	nd	nd
Q-3-hex	7.37	463;301	nd	nd	3.96 (0–11.89)	17.04 (0–28.70)	5.35 (0–16.05)	30.80 (18.40–43.20)
M-3-gcn	8.36	493;317	nd	2.99 (0–26.94)	nd	nd	nd	nd
Dq-3-hex	9.50	465;303	14.69 (0–29.37)	2.74 (0–13.82)	5.76 (0–17.27)	6.32 (0–12.80)	2.96 (0–8.87)	nd
M-3-gal	12.44	479;317	nd	6.22 (0–55.95)	4.88 (0–14.63)	2.83 (0–11.34)	nd	nd
M-3-glc	13.45	479,317	20.37 (19.78–20.96)	28.38 (0–88.99)	36.00 (29.31–47.20)	8.02 (0–19.58)	13.15 (0–20.50)	34.31 (30.12–38.50)
M-3-rha	16.34	463;317	nd	6.32 (0–38.61)	nd	9.43 (0–37.72)	nd	215.85 (193.38–238.31)
Q	16.61	301	nd	36.24 (0–147.55)	54.38 (0–129.12)	23.82 (0–95.29)	36.13 (0–69.21)	505.02 (452.60–557.43)
Dq-3-rha	17.70	449;303	53.46 (30.23–76.70)	nd	nd	19.83 (0–69.93)	nd	nd
Q-3-gal	18.14	463;301	19.00 (11.94–26.06)	7.62 (0–39.09)	6.50 (0–19.49)	4.10 (0–16.40)	6.49 (0–19.46)	nd
Q-3-gcn	19.23	477;301	39.82 (33.32–46.33)	60.26 (11.84–166.95)	77.76 (37.42–141.04)	154.05 (88.97–212.12)	121.33 (35.54–272.54)	nd
Q-3-glc	19.79	463;301	85.86 (56.66–115.06)	57.65 (16.92–155.21)	50.19 (25.75–85.48)	170.01 (68.01–369.49)	69.81 (7.71–166.18)	nd
Q-3-rut	20.03	609;301	nd	2.06 (0–18.58)	74.33 (0–223.00)	74.61 (0–278.92)	39.66 (0–100.95)	nd
Q-3-rha	20.84	447,301	nd	111.70 (0–528.65)	nd	66.07 (0–176.20)	5.04 (0–15.13)	652.76 (630.48–675.03)
L-3-glc	21.92	493;331	20.04 (16.55–23.53)	3.85 (0–15.87)	6.34 (0–19.02)	3.30 (0–13.21)	8.07 (0–12.96)	nd
K-3-gal	22.27	447;285	6.83 (0–13.67)	nd	nd	nd	nd	13.33 (0–26.66)
Q-3-xyl	22.30	433;301	nd	12.49 (0–47.22)	13.88 (0–41.65)	8.49 (0–33.98)	14.17 (0–26.25)	244.04 (240.55–247.53)
I-3-xyl	22.57	447;315	nd	2.67 (0–24.03)	nd	10.05 (0–40.20)	nd	14.15 (0–28.30)
K-3-rha	22.74	431;285	nd	8.17 (0–57.18)	nd	nd	nd	63.87 (63.09–64.65)
Dk-3-rha	24.28	433;287	nd	2.96 (0–26.68)	nd	4.43 (0–17.70)	nd	nd
K-3-glc	25.58	447;285	51.34 (0–102.68)	nd	nd	nd	nd	20.89 (15.69–26.09)
L-3-acglc	28.13	535;331	nd	12.45 (0–62.68)	8.13 (0–24.40)	nd	nd	nd
I-3-glc	29.16	477;315	85.77 (0–171.53)	5.28 (0–21.23)	4.07 (0–12.22)	11.34 (0–33.31)	nd	nd
I-3-rha	30.24	461;315	nd	1.19 (0–10.68)	nd	nd	nd	13.08 (0–26.16)
S-3-glc	30.45	507;345	66.60 (63.35–69.85)	13.58 (0–63.58)	nd	11.25 (0–27.28)	5.32 (0–15.97)	nd
Dq-3-acglc	31.07	627;465,303	nd	nd	nd	5.89 (0–23.57)	14.94 (0–44.81)	nd
K-3-rut	36.88	539;285	nd	1.10 (0–9.88)	58.62 (0–175.86)	138.93 (0–475.01)	33.51 (0–100.54)	nd

*Flavan-3-ols (μg CE/g DW)*								
Gallocatechin	0.91	305;179,137	4.82 (0–9.64)	nd	nd	nd	nd	18.03 (15.34–20.72)
Epigallocatechin	2.33	305;179,141	nd	nd	nd	nd	nd	27.25 (21.05–33.45)
Catechin	2.87	289	22.01 (9.90–34.13)	2.49 (0–22.42)	nd	6.42 (0–25.68)	13.92 (0–25.90)	32.85 (0–65.71)
Epicatechin	6.44	289	6.54 (0–13.09)	8.39 (0–28.66)	nd	nd	nd	223.78 (163.53–284.03)
Procyanidin dimmer 1	2.18	577;425,289	62.05 (50.99–73.11)	44.52 (0–381.80)	187.58 (0–562.73)	342.42 (6.45–1198.00)	70.26 (0–154.99)	72.41 (31.55–113.28)
Procyanidin dimmer 2	5.21	577;425,289	6.06 (0–12.12)	nd	nd	nd	nd	26.72 (0–53.44)
Procyanidin dimmer 3	10.98	577;425,289	29.11 (0–58.22)	nd	nd	nd	nd	nd
Procyanidin trimer	4.16	865; 577,289	4.77 (0–9.53)	nd	nd	11.42 (0–45.67)	nd	nd

nd means not detected. tr means trace. Abbreviations: Eu, European; As, East Asian; Am, North Amrican; Mu, Muscadine; Q, quercetin; K, kaempferol; Ir, isorhamnetin; L, laricitrin; S, syringetin; Dq, dihydroquercetin; Dk, dihydrokaempferol; gal, galactoside; gcn, glucuronide; rha, rhamnoside; caglc, (6-caffeoyl)-glucoside; hex, hexoside; xyl, xyloside; rut, rutinoside. The contents of the dihydroflavonol, flavonol and flavan-3-ol compounds in the skins of each wine grape cultivars are shown in the supplementary file.

**Table 5:** Retention times, ESI-MS/MS *m*/*z* values (molecular ion (MS); product ions (MS^2^)), average contents and range in parentheses of nonflavonoid compounds in the skins of different grape groups.

Compounds	Rt (min)	MS; MS^2^ (*m*/*z*)	Eu-grapes	As-grapes	Eu-As hybrids	Am-grapes	Eu-Am hybrids	Mu-grapes
*Cinnamic acids (μg CAE/g DW)*								
Chlorgenic acid	0.56	191	0.67 (0–1.33)	7.57 (0–10.16)	5.83 (0–9.82)	6.13 (0.38–8.85)	4.86 (0.71–7.09)	8.07 (7.89–8.26)
Caffeic acid	1.19	179	nd	1.15 (0–10.36)	nd	1.63 (0–5.55)	nd	nd
Caftaric acid	1.30	311;179	nd	8.56 (0–24.16)	nd	nd	nd	nd
*p*-Coumaric acid	2.90	163	nd	1.68 (0–11.45)	3.28 (0–9.85)	2.25 (0–8.99)	7.92 (0–23.75)	7.40 (0–14.80)
Ferulic acid	3.01	193	nd	15.36 (0–58.44)	17.81 (0–36.13)	12.40 (0–32.58)	5.13 (0–7.87)	nd
HE of caffeic acid	5.63	341;179	nd	nd	nd	nd	nd	17.64 (0–35.29)
HE of *p*-coumaric acid	6.11	325;163	nd	1.82 (0–16.36)	nd	nd	3.26 (0–9.79)	4.15 (0–8.29)
HE of ferulic acid	7.47	355;193	0 (0-tr)	16.98 (0–131.33)	69.31 (10.26–183.29)	13.18 (6.64–26.55)	4.15 (1.25–8.97)	4.17 (0–8.34)
Fertaric acid	8.45	325;193	nd	58.15 (0–196.63)	11.87 (0–25.11)	8.50 (0–27.86)	7.21 (0–18.15)	nd

*Benzoic acids (μg GAE/g DW)*								
HE of protocatechuic acid	0.86	315;153	0.46 (0–0.91)	6.96 (3.10–12.59)	4.84 (tr-11.51)	0.05 (0–0.19)	4.38 (0–8.08)	9.20 (7.07–11.34)
protocatechuic acid	1.41	153	nd	0.17 (0–1.56)	nd	nd	nd	nd
*p*-Hydroxybenzoic acid	1.77	137	nd	0.79 (0–3.70)	nd	nd	nd	nd
Ethyl gallate	4.41	197;169	nd	1.45 (0–13.05)	nd	0.94 (0–3.78)	3.55 (0–6.08)	nd
HE of vanillic acid	5.15	329;167	8.95 (7.73–10.17)	3.99 (0–15.27)	7.63 (4.39–11.17)	6.75 (0.58–12.73)	3.70 (tr-6.30)	8.36 (6.96–9.75)

*Ellagic acids (μg EAE/g DW)*								
Ellagic acid-rha	9.33	331;169,125	nd	nd	nd	nd	nd	16.49 (0–32.97)
HHDP-galloylglucose	0.64	633;481,301	nd	nd	nd	nd	nd	140.16 (95.42–184.91)
HHDP-glucose	3.81	481;421,301	nd	nd	nd	nd	nd	5.41 (0.00–10.82)
Ellagitannin 1	9.76	813;781,301	nd	nd	nd	nd	nd	92.91 (92.37–93.45)
Ellagitannin 2	14.47	831;813,301	nd	nd	nd	nd	nd	331.45 (303.32–359.59)

*Stilbenes (μg RE/g DW)*								
*trans*-Piceid	10.19	389;227	2.50 (tr–5.01)	0.96 (0–8.67)	1.21 (0–3.64)	nd	nd	28.21 (tr-56.41)
*trans*-Resveratrol	23.67	227	94.44 (26.83–162.05)	10.25 (0–67.09)	9.10 (tr-27.31)	0 (0-tr)	0 (0-tr)	13.71 (12.15–15.26)

nd means not detected. tr means trace. Abbreviations: Eu, European; As, East Asian; Am, North Amrican; Mu, Muscadine; HE, hexose ester; rha, rhamnoside. The contents of the nonflavonoid compounds in the skins of each wine grape cultivars are shown in the supplementary file.
