# Supplementary material for: Phenolic Contents and Compositions in Skins of Red Wine Grape Cultivars among Various Genetic Backgrounds and Originations
Source: Int J Mol Sci. 2012 Mar 14;13(3):3492–510. doi: 10.3390/ijms13033492 (PMC3317724; doi:10.3390/ijms13033492)
Supplement: Supplementary file 1 [file ijms-13-03492-s001.pdf]

Supplementary Information

Appendix 1. Contents of anthocyanin compounds in the skins of different grape cultivars

Cultivars [c] / Compounds [a,b] (µg MGE/g DW)	CS	ML	CB	SH	SY	ZS-1	ZS-2	BP	MA	MI	SN	HS	ZH
Dp-3,5-diglc	nd	nd	656.55 ± 6.64	1290.08 ± 17.29	440.79 ± 12.95	1552.50 ± 180.73	1704.53 ± 8.41	nd	nd	44.87 ± 3.60	2378.51 ± 19.10	106.45 ± 9.36	742.17 ± 7.50
Cy-3,5-diglc	nd	nd	373.07 ± 5.02	224.25 ± 5.41	1008.01 ± 15.97	601.53 ± 15.45	1143.16 ± 16.80	nd	nd	nd	392.48 ± 10.14	14.46 ± 1.56	322.82 ± 8.21
Pt-3,5-diglc	nd	nd	630.15 ± 6.41	1036.74 ± 15.43	600.44 ± 17.39	1257.30 ± 23.38	1340.57 ± 7.03	43.14 ± 5.63	33.51 ± 4.33	51.34 ± 6.70	2734.42 ± 20.04	33.73 ± 3.89	437.13 ± 8.35
Dp-3-glc	123.60 ± 45.26	123.18 ± 16.16	367.04 ± 13.10	277.11 ± 12.49	241.39 ± 9.35	878.16 ± 17.31	2634.50 ± 9.96	nd	53.06 ± 5.43	25.95 ± 2.54	979.97 ± 17.11	2719.36 ± 18.08	5679.48 ± 8.11
Pg-3,5-diglc	nd	nd	nd	nd	nd	nd	nd	nd	nd	nd	nd	nd	nd
Pn-3,5-diglc	nd	nd	335.97 ± 9.19	478.22 ± 15.97	508.73 ± 12.51	766.37 ± 15.17	897.11 ± 6.45	84.20 ± 5.07	37.87 ± 3.70	74.78 ± 3.44	562.62 ± 13.72	41.84 ± 5.76	291.08 ± 4.02
Mv-3,5-diglc	nd	nd	2150.57 ± 17.06	3105.77 ± 20.08	1554.45 ± 40.22	4034.56 ± 37.96	4663.20 ± 9.27	794.17 ± 14.20	133.01 ± 7.93	758.51 ± 9.26	4148.34 ± 22.49	53.16 ± 6.83	2547.98 ± 6.55
Cy-3-glc	47.21 ± 3.81	48.37 ± 6.86	nd	nd	nd	nd	nd	nd	nd	nd	nd	593.03 ± 10.69	nd
Pt-3-glc	127.97 ± 28.72	116.90 ± 7.36	78.45 ± 7.45	108.71 ± 8.74	44.52 ± 3.68	522.61 ± 4.77	748.27 ± 5.16	nd	57.44 ± 2.87	nd	194.02 ± 7.70	1548.03 ± 19.75	2153.69 ± 8.30
Pg-3-glc	nd	nd	nd	nd	nd	nd	nd	nd	nd	nd	nd	nd	nd
Cy-3-acglc-5-glc	nd	nd	nd	nd	nd	nd	nd	nd	tr	nd	nd	nd	nd
Pt-3-acglc-5-glc	nd	nd	nd	nd	nd	nd	nd	nd	nd	nd	nd	nd	nd
Pn-3-glc	263.83 ± 22.08	218.63 ± 27.18	13.12 ± 2.03	10.33 ± 0.90	53.36 ± 7.10	43.43 ± 5.42	272.50 ± 6.94	nd	25.33 ± 1.52	19.13 ± 3.39	26.09 ± 7.90	69.58 ± 4.72	268.92 ± 6.60

Appendix 1. *Cont.*

Compounds [a,b] (μg MGE/g DW) \ Cultivars [c]	CS	ML	CB	SH	SY	ZS-1	ZS-2	BP	MA	MI	SN	HS	ZH
Mv-3-glc	2153.27 ± 35.26	1436.27 ± 204.25	51.77 ± 5.36	63.92 ± 7.32	27.49 ± 6.11	380.50 ± 23.47	967.11 ± 14.98	27.38 ± 5.31	149.43 ± 8.28	32.78 ± 4.78	124.03 ± 6.25	411.91 ± 7.56	1458.67 ± 5.26
Dp-3-cfglc-5-glc	nd	nd	nd	nd	nd	nd	nd	nd	nd	nd	nd	nd	nd
Dp-3-cis-cmglc-5-glc	nd	nd	nd	nd	nd	nd	nd	nd	nd	nd	nd	nd	nd
Pn-3-acglc-5-glc	nd	nd	nd	nd	nd	nd	nd	nd	nd	nd	nd	nd	nd
Dp-3-acglc	nd	nd	nd	nd	nd	nd	nd	nd	nd	nd	nd	nd	nd
Mv-3-acglc-5-glc	nd	nd	nd	nd	nd	nd	nd	7.42 ± 0.38	tr	24.29 ± 2.97	7.44 ± 0.35	nd	nd
Dp-3-trans-cmglc-5-glc	nd	nd	nd	nd	nd	nd	nd	16.54 ± 1.86	11.07 ± 0.94	nd	11.84 ± 1.39	226.08 ± 12.51	nd
Cy-3-acglc	nd	nd	nd	nd	nd	nd	nd	nd	nd	nd	nd	nd	nd
Dp-3-cfglc	nd	nd	nd	nd	nd	nd	nd	nd	6.07 ± 0.37	nd	nd	65.57 ± 9.02	nd
Pt-3-cis-cmglc-5-glc	nd	nd	nd	nd	nd	nd	nd	nd	7.30 ± 0.52	nd	nd	14.90 ± 1.95	nd
Pt-3-acglc	nd	nd	nd	nd	nd	nd	nd	nd	tr	nd	nd	65.58 ± 8.04	nd
Dp-3-cis-cmglc	nd	nd	nd	nd	nd	nd	nd	nd	9.86 ± 0.95	nd	nd	nd	nd
Cy-3-cmglc-5-glc	nd	nd	nd	nd	nd	nd	nd	nd	nd	nd	nd	61.05 ± 5.50	nd
Pt-3-trans-cmglc-5-glc	nd	nd	nd	nd	nd	nd	nd	40.05 ± 6.56	22.48 ± 2.48	nd	nd	177.25 ± 10.92	nd
Mv-3-cfglc-5-glc	nd	nd	nd	nd	nd	nd	nd	nd	16.37 ± 1.16	79.97 ± 8.38	nd	nd	nd

Appendix 1. *Cont.*

Cultivars [c] / Compounds [a,b] (μg MGE/g DW)	CS	ML	CB	SH	SY	ZS-1	ZS-2	BP	MA	MI	SN	HS	ZH
Pt-3-cfglc	nd	nd	nd	nd	nd	nd	nd	nd	11.20 ± 0.82	nd	nd	nd	nd
Pn-3-cis-cmglc-5-glc	nd	nd	nd	nd	nd	nd	nd	nd	tr	nd	nd	nd	nd
Mv-3-cis-cmglc-5-glc	nd	nd	nd	nd	nd	nd	nd	19.68 ± 2.78	24.03 ± 2.42	18.16 ± 2.83	nd	nd	nd
Dp-3-trans-cmglc	nd	nd	nd	nd	nd	nd	nd	nd	67.26 ± 7.16	tr	13.64 ± 1.16	1170.68 ± 14.49	nd
Pn-3-acglc	60.67 ± 23.44	51.03 ± 1.53	nd	nd	nd	nd	nd	nd	nd	nd	nd	nd	nd
Mv-3-acglc	607.68 ± 57.68	414.43 ± 8.93	nd	nd	nd	nd	nd	nd	18.51 ± 1.97	nd	nd	30.07 ± 3.85	nd
Cy-3-cis-cmglc	nd	nd	nd	nd	nd	nd	nd	nd	nd	nd	nd	nd	nd
Pn-3-trans-cmglc-5-glc	nd	nd	nd	nd	nd	nd	nd	nd	nd	nd	nd	nd	nd
Mv-3-trans-cmglc-5-glc	nd	nd	nd	nd	nd	nd	nd	522.16 ± 14.01	147.97 ± 8.64	583.23 ± 8.37	nd	56.41 ± 5.06	nd
Pt-3-cis-cmglc	nd	nd	nd	nd	nd	nd	nd	nd	8.36 ± 0.58	nd	nd	nd	nd
Mv-3-cfglc	49.85 ± 4.30	26.26 ± 1.88	nd	nd	nd	nd	nd	nd	12.96 ± 1.40	nd	nd	143.90 ± 11.86	nd
Cy-3-trans-cmglc	nd	nd	nd	nd	nd	nd	nd	nd	16.38 ± 1.31	nd	nd	7.74 ± 0.45	nd
Pt-trans-cmglc	69.43 ± 11.63	75.78 ± 4.75	nd	nd	nd	nd	nd	nd	75.70 ± 5.04	9.76 ± 0.90	nd	344.38 ± 5.20	nd
Pt-3-cis-cmglc	nd	nd	nd	nd	nd	nd	nd	nd	tr	nd	nd	nd	nd

Appendix 1. *Cont.*

Compounds [a,b] (μg MGE/g DW) \ Cultivars [c]	CS	ML	CB	SH	SY	ZS-1	ZS-2	BP	MA	MI	SN	HS	ZH
Mv-3-cis-cmglc	nd	nd	nd	nd	nd	nd	nd	nd	22.12 ± 5.10	nd	nd	nd	nd
Pt-3-trans-cmglc	93.57 ± 11.48	102.76 ± 13.74	nd	nd	nd	nd	nd	tr	219.00 ± 9.96	8.38 ± 0.96	tr	10.97 ± 0.80	nd
Mv-3-trans-cmglc	566.21 ± 80.04	544.50 ± 74.54	nd	nd	nd	nd	nd	52.69 ± 6.51	6.02 ± 0.43	70.23 ± 6.24	7.17 ± 0.35	63.23 ± 6.29	nd
Total [d]	4163.28 ± 151.49 [e,f]	3158.11 ± 323.03 [d]	4656.70 ± 72.26 [g]	6595.13 ± 103.62 [i]	4479.19 ± 125.29 [f,g]	10036.96 ± 113.72 [m]	14370.94 ± 16.04 [o]	1607.43 ± 62.31 [b,c]	1192.31 ± 85.32 [a,b]	1801.37 ± 64.34 [c]	11580.58 ± 127.70 [n]	8029.36 ± 184.13 [k]	13901.93 ± 47.90 [o]

Compounds [a,b] (μg MGE/g DW) \ Cultivars [c]	ZY	BS	CT	CC	NR	MF	CH	SC	AL	NB
Dp-3,5-diglc	839.73 ± 10.16	644.79 ± 6.59	42.63 ± 3.68	170.39 ± 14.63	nd	89.48 ± 4.32	191.60 ± 6.88	1038.96 ± 10.72	1318.86 ± 7.03	533.73 ± 15.56
Cy-3,5-diglc	452.11 ± 9.38	272.07 ± 7.73	83.65 ± 4.31	207.50 ± 14.23	102.58 ± 4.44	29.23 ± 2.30	61.99 ± 3.75	529.53 ± 6.72	563.84 ± 3.94	2006.81 ± 7.69
Pt-3,5-diglc	474.79 ± 12.39	499.54 ± 9.01	22.58 ± 3.22	105.44 ± 8.48	nd	35.66 ± 3.14	326.56 ± 12.00	1426.36 ± 17.89	653.75 ± 2.57	1137.16 ± 8.40
Dp-3-glc	505.87 ± 12.89	673.31 ± 7.50	449.41 ± 3.42	1137.04 ± 21.19	141.58 ± 1.32	343.65 ± 4.97	740.65 ± 5.69	2383.92 ± 85.80	nd	nd
Pg-3,5-diglc	nd	nd	nd	nd	nd	nd	nd	nd	31.59 ± 0.92	162.77 ± 6.22
Pn-3,5-diglc	429.82 ± 7.37	568.77 ± 6.19	43.61 ± 2.29	167.92 ± 11.13	nd	36.16 ± 5.77	251.64 ± 4.40	498.54 ± 8.57	190.00 ± 5.91	1166.33 ± 7.24
Mv-3,5-diglc	1497.23 ± 10.12	1277.69 ± 11.36	nd	192.40 ± 8.20	nd	268.32 ± 8.62	2603.58 ± 13.62	2950.67 ± 14.55	162.06 ± 7.26	542.11 ± 10.93

Appendix 1. *Cont.*

Cultivars[c] / Compounds[a,b] (μg MGE/g DW)	ZY	BS	CT	CC	NR	MF	CH	SC	AL	NB
Cy-3-glc	nd	40.07 ± 7.30	643.38 ± 4.28	608.31 ± 20.59	741.42 ± 7.03	nd	nd	nd	nd	nd
Pt-3-glc	96.60 ± 4.76	231.98 ± 14.92	57.04 ± 2.46	252.17 ± 11.44	6.12 ± 0.68	404.02 ± 8.48	891.46 ± 8.38	1105.79 ± 1.52	nd	nd
Pg-3-glc	nd	47.45 ± 2.37	nd	nd	9.50 ± 0.31	nd	nd	nd	nd	nd
Cy-3-acglc-5-glc	nd	nd	nd	nd	nd	7.08 ± 0.12	nd	nd	nd	nd
Pt-3-acglc-5-glc	nd	62.39 ± 0.30	nd	nd	nd	nd	nd	140.13 ± 0.59	nd	nd
Pn-3-glc	29.55 ± 2.99	134.85 ± 4.75	43.21 ± 1.71	93.29 ± 9.89	12.70 ± 0.19	39.56 ± 6.73	93.16 ± 8.18	106.07 ± 5.37	nd	nd
Mv-3-glc	95.39 ± 8.40	201.12 ± 5.90	18.39 ± 0.57	92.96 ± 9.42	nd	1160.49 ± 5.49	1634.41 ± 13.57	488.69 ± 2.10	nd	nd
Dp-3-cfglc-5-glc	nd	79.81 ± 6.54	nd	nd	nd	nd	nd	nd	nd	nd
Dp-3-cis-cmglc-5-glc	nd	30.24 ± 0.84	nd	91.26 ± 7.58	nd	nd	nd	nd	nd	nd
Pn-3-acglc-5-glc	nd	nd	nd	nd	nd	nd	nd	nd	nd	nd
Dp-3-acglc	nd	73.15 ± 1.16	nd	nd	nd	34.04 ± 2.79	52.73 ± 0.11	169.32 ± 2.98	nd	nd
Mv-3-acglc-5-glc	nd	60.69 ± 1.71	nd	nd	nd	40.41 ± 6.05	27.98 ± 2.54	91.20 ± 1.17	nd	nd
Dp-3-trans-cmglc-5-glc	nd	1304.32 ± 2.52	nd	319.62 ± 15.03	nd	18.45 ± 0.28	132.03 ± 1.27	1666.21 ± 26.63	nd	nd
Cy-3-acglc	nd	nd	nd	42.15 ± 6.18	nd	nd	nd	nd	nd	nd
Dp-3-cfglc	nd	nd	nd	nd	nd	nd	nd	nd	nd	nd
Pt-3-cis-cmglc-5-glc	nd	55.27 ± 2.48	nd	8.64 ± 0.55	nd	nd	26.62 ± 3.01	nd	nd	nd
Pt-3-acglc	nd	45.40 ± 0.33	nd	45.33 ± 2.82	nd	45.55 ± 2.58	35.50 ± 0.98	77.05 ± 6.06	nd	nd
Dp-3-cis-cmglc	nd	nd	nd	nd	nd	nd	nd	nd	nd	nd
Cy-3-cmglc-5-glc	nd	336.70 ± 5.85	nd	327.50 ± 13.16	nd	13.57 ± 0.86	nd	289.80 ± 3.93	nd	nd
Pt-3-trans-cmglc-5-glc	nd	691.57 ± 6.55	nd	132.72 ± 9.49	nd	46.92 ± 3.35	192.26 ± 5.41	973.12 ± 25.51	nd	nd
Mv-3-cfglc-5-glc	nd	nd	nd	nd	nd	nd	nd	nd	nd	nd
Pt-3-cfglc	nd	nd	nd	nd	nd	10.43 ± 0.45	24.90 ± 1.38	15.36 ± 0.65	nd	nd
Pn-3-cis-cmglc-5-glc	nd	21.84 ± 1.05	nd	11.97 ± 0.49	nd	nd	nd	nd	nd	nd
Mv-3-cis-cmglc-5-glc	nd	nd	nd	nd	nd	nd	nd	48.24 ± 3.68	nd	nd
Dp-3-trans-cmglc	nd	462.59 ± 3.95	10.15 ± 0.35	798.26 ± 24.93	7.03 ± 0.55	38.37 ± 6.21	232.28 ± 7.65	1456.98 ± 3.10	nd	nd
Pn-3-acglc	nd	nd	nd	92.07 ± 8.73	nd	10.65 ± 0.36	nd	nd	nd	nd
Mv-3-acglc	nd	33.86 ± 3.21	nd	15.90 ± 1.45	nd	114.01 ± 4.30	29.44 ± 3.35	15.20 ± 0.64	nd	nd

Appendix 1. *Cont.*

Cultivars[c] / Compounds[a,b] (μg MGE/g DW)	ZY	BS	CT	CC	NR	MF	CH	SC	AL	NB
Cy-3-cis-cmglc	nd	nd	nd	13.49 ± 0.91	7.88 ± 0.49	nd	nd	nd	nd	nd
Pn-3-trans-cmglc-5-glc	nd	328.97 ± 8.15	nd	90.20 ± 6.09	nd	nd	nd	nd	nd	nd
Mv-3-trans-cmglc-5-glc	nd	391.45 ± 6.03	nd	103.67 ± 8.83	nd	45.62 ± 1.12	297.72 ± 4.43	702.93 ± 7.93	nd	nd
Pt-3-cis-cmglc	nd	nd	nd	nd	nd	nd	nd	nd	nd	nd
Mv-3-cfglc	nd	104.37 ± 6.40	36.06 ± 2.43	454.79 ± 4.92	36.82 ± 2.28	10.99 ± 0.82	26.38 ± 2.47	157.78 ± 5.26	nd	nd
Cy-3-trans-cmglc	nd	nd	nd	nd	nd	20.85 ± 1.17	39.02 ± 6.10	nd	nd	nd
Pt-trans-cmglc	nd	92.60 ± 5.10	nd	132.89 ± 9.72	nd	28.41 ± 2.09	200.85 ± 8.00	346.48 ± 6.48	nd	nd
Pt-3-cis-cmglc	nd	nd	nd	nd	nd	nd	nd	nd	nd	nd
Mv-3-cis-cmglc	nd	nd	nd	nd	nd	nd	13.12 ± 1.31	nd	nd	nd
Pt-3-trans-cmglc	nd	20.70 ± 0.58	6.92 ± 0.25	37.45 ± 4.89	nd	7.98 ± 0.39	11.74 ± 1.14	13.14 ± 0.23	nd	nd
Mv-3-trans-cmglc	nd	55.25 ± 0.39	tr	47.69 ± 4.24	nd	49.41 ± 4.95	313.01 ± 13.27	149.52 ± 8.51	nd	nd
Total[d]	4421.08 ± 78.47[f,g]	8842.82 ± 18.65[l]	1457.04 ± 13.47[a,b,c]	5792.99 ± 258.15[h]	1065.63 ± 12.84[a]	2949.30 ± 87.68[d]	8450.64 ± 20.74[k,l]	16840.99 ± 60.59[p]	2920.11 ± 25.80[d]	5548.91 ± 6.36[h]

nd means not detected. tr means trace. Abbreviations: dp, delphinidin; cy, cyanidin; pt, petunidin; pg, pelargonidin; pn, peonidin; mv, malvidin; glc, glucoside; diglc, diglucoside; acglc, (6-acetyl)-glucoside; cfglc, (6-caffeoyl)-glucoside; cmglc, (6-coumaroyl)-glucoside; [a] Each number represents an anthocyanin compound, see appendix 2; [b] Values are means of duplicate determination ± S.D.; [c] Each abbreviation represents a cultivar, see Table 1; [d] Different letters in each column are significantly different at 0.05 level from ANOVA.

Appendix 2. Contents of non-anthocyanin phenolic compounds in the skins of different grape cultivars.

Cultivars [c] / Compounds [a,b]	CS	ML	CB	SH	SY	ZS-1	ZS-2	BP	MA	MI	SN	HS	ZH
						Flavonols and dihydroflavonols (μg QE/g DW)							
Dk-3-glc	nd	nd	nd	nd	nd	nd	nd	nd	nd	nd	nd	nd	nd
K-3-hex	nd	nd	nd	nd	nd	nd	nd	nd	nd	nd	nd	nd	nd
Q-3-hex	nd	nd	nd	nd	nd	nd	nd	nd	nd	nd	nd	nd	11.89 ± 2.64
M-3-gcn	nd	nd	nd	nd	nd	nd	nd	nd	nd	nd	26.94 ± 1.73	nd	nd
Dq-3-hex	nd	29.37 ± 1.21	13.82 ± 0.20	nd	nd	nd	nd	nd	nd	10.85 ± 1.37	nd	nd	17.27 ± 2.63
M-3-gal	nd	nd	nd	nd	nd	nd	nd	nd	nd	nd	55.95 ± 3.81	nd	14.63 ± 1.74
M-3-glc	19.78 ± 3.84	20.96 ± 10.73	13.96 ± 0.59	41.44 ± 0.24	nd	73.05 ± 1.57	14.10 ± 0.14	nd	23.91 ± 0.16	nd	88.99 ± 5.09	29.31 ± 4.31	31.51 ± 1.70
M-3-rha	nd	nd	18.30 ± 0.73	nd	nd	nd	nd	nd	nd	nd	38.61 ± 10.04	nd	nd
Q	nd	nd	147.55 ± 7.59	40.84 ± 0.79	nd	43.15 ± 1.31	23.99 ± 0.13	nd	nd	nd	70.67 ± 11.86	nd	129.12 ± 2.72
Dq-3-rha	30.23 ± 0.33	76.70 ± 0.92	nd	nd	nd	nd	nd	nd	nd	nd	nd	nd	nd
Q-3-gal	26.06 ± 0.08	11.94 ± 4.37	16.54 ± 0.37	12.94 ± 0.44	nd	nd	nd	nd	nd	nd	39.09 ± 4.55	nd	nd
Q-3-gcn	46.33 ± 2.70	33.32 ± 3.63	108.79 ± 2.95	26.50 ± 0.10	48.08 ± 1.44	37.39 ± 0.02	36.90 ± 0.44	16.13 ± 0.52	166.95 ± 4.89	11.84 ± 0.26	89.79 ± 6.91	141.04 ± 22.05	37.42 ± 2.58
Q-3-glc	115.06 ± 1.81	56.66 ± 2.07	120.00 ± 1.91	77.66 ± 0.38	19.00 ± 1.59	38.68 ± 0.61	38.30 ± 0.10	20.53 ± 0.64	32.56 ± 0.27	16.92 ± 0.11	155.21 ± 5.57	25.75 ± 5.19	39.35 ± 4.19
Q-3-rut	nd	nd	nd	nd	nd	nd	nd	nd	nd	nd	18.58 ± 2.80	223.00 ± 8.15	nd
Q-3-rha	nd	nd	nd	nd	nd	nd	nd	271.28 ± 1.64	26.60 ± 0.97	178.80 ± 3.71	528.65 ± 23.88	nd	nd

Appendix 2. *Cont.*

Compounds [a,b] / Cultivars [c]	CS	ML	CB	SH	SY	ZS-1	ZS-2	BP	MA	MI	SN	HS	ZH
Flavonols and dihydroflavonols (μg QE/g DW)													
L-3-glc	16.55 ± 0.19	23.53 ± 0.93	nd	nd	nd	nd	nd	9.99 ± 0.26	nd	8.81 ± 0.35	15.87 ± 4.80	19.02 ± 1.85	nd
K-3-gal	13.67 ± 1.57	nd	nd	nd	nd	nd	nd	nd	nd	nd	nd	nd	nd
Q-3-xyl	nd	nd	nd	nd	nd	nd	nd	14.79 ± 0.02	47.22 ± 1.22	12.41 ± 1.33	38.01 ± 6.00	nd	41.65 ± 2.30
I-3-xyl	nd	nd	nd	nd	nd	nd	nd	nd	nd	nd	24.03 ± 6.27	nd	nd
K-3-rha	nd	nd	nd	nd	nd	nd	nd	tr	16.34 ± 0.48	nd	57.18 ± 9.97	nd	nd
Dk-3-rha	nd	nd	nd	nd	nd	nd	nd	nd	nd	nd	26.68 ± 6.58	nd	nd
K-3-glc	102.68 ± 26.00	nd	nd	nd	nd	nd	nd	nd	nd	nd	nd	nd	nd
L-3-acglc	nd	nd	nd	nd	nd	nd	nd	nd	49.37 ± 4.76	nd	62.68 ± 9.77	nd	24.40 ± 0.73
I-3-glc	171.53 ± 35.68	nd	nd	nd	nd	nd	nd	13.09 ± 0.71	nd	13.16 ± 2.98	21.23 ± 3.42	nd	12.22 ± 0.62
I-3-rha	nd	nd	nd	nd	nd	nd	nd	nd	10.68 ± 0.12	nd	nd	nd	nd
S-3-glc	69.85 ± 23.60	63.35 ± 24.11	nd	nd	nd	nd	nd	46.55 ± 3.25	12.10 ± 0.67	63.58 ± 8.26	nd	nd	nd
Dq-3-acglc	nd	nd	nd	nd	nd	nd	nd	nd	nd	nd	nd	nd	nd
K-3-rut	nd	nd	nd	nd	nd	nd	nd	nd	nd	9.88 ± 0.31	nd	nd	nd
Tota l [d]	611.75 ± 39.84 [g,h,i]	315.83 ± 41.98 [b,c,d]	438.97 ± 13.61 [e,f,g,h]	199.3 ± 1.75 [a,b,c,d]	67.08 ± 3.03 [a]	192.27 ± 2.26 [a,b,c,d]	113.29 ± 0.36 [a,b]	392.36 ± 0.88 [d,e,f,g]	385.74 ± 11.02 [d,e,f]	326.26 ± 18.68 [b,c,d,e]	1358.17 ± 123.03 [k]	613.97 ± 48.46 [h,i]	359.46 ± 15.11 [c,d,e]

Appendix 2. *Cont.*

Compounds [a,b] / Cultivars [c]	CS	ML	CB	SH	SY	ZS-1	ZS-2	BP	MA	MI	SN	HS	ZH
Flavan-3-ols (μg CE/g DW)													
Gallocatechin	nd	9.64 ± 0.08	nd	nd	nd	nd	nd	nd	nd	nd	nd	nd	nd
Epigallocatechin	nd	nd	nd	nd	nd	nd	nd	nd	nd	nd	nd	nd	nd
Catechin	9.90 ± 0.30	34.13 ± 1.69	nd	nd	nd	nd	nd	nd	nd	nd	22.42 ± 1.22	nd	nd
Epicatechin	nd	13.09 ± 0.16	nd	nd	nd	nd	nd	16.46 ± 1.14	16.08 ± 7.05	14.30 ± 4.83	28.66 ± 6.40	nd	nd
Procyanidin dimer 1	50.99 ± 1.27	73.11 ± 27.48	381.80 ± 12.38	nd	nd	nd	nd	nd	nd	nd	18.88 ± 2.55	562.73 ± 308.23	nd
Procyanidin dimer 2	nd	12.12 ± 0.13	nd	nd	nd	nd	nd	nd	nd	nd	nd	nd	nd
Procyanidin dimer 3	nd	58.22 ± 22.25	nd	nd	nd	nd	nd	nd	nd	nd	nd	nd	nd
Procyanidin trimer	nd	9.53 ± 0.85	nd	nd	nd	nd	nd	nd	nd	nd	nd	nd	nd
Total [d]	60.89 ± 0.97 [a]	209.84 ± 47.39 [a,b,c,d]	381.80 ± 12.38 [c,d,e]	nd	nd	nd	nd	16.46 ± 1.14[a]	16.08 ± 7.05 [a]	14.30 ± 4.83 [a]	69.96 ± 2.62 [a]	562.73 ± 308.23 [e]	nd
Cinnamic acids (μg CAE/g DW)													
Chlorgenic acid	1.33 ± 0.00	nd	8.16 ± 0.08	10.16 ± 0.45	10.00 ± 0.28	8.17 ± 0.15	8.04 ± 0.14	7.43 ± 0.03	10.04 ± 0.03	6.09 ± 0.03	nd	9.82 ± 0.86	7.67 ± 0.16
Caffeic acid	nd	nd	nd	nd	10.36 ± 0.33	nd	nd	nd	nd	nd	nd	nd	nd
Caftaric acid	nd	nd	16.40 ± 0.99	nd	nd	nd	nd	22.65 ± 0.12	24.16 ± 0.07	13.86 ± 0.10	nd	nd	nd

Appendix 2. *Cont.*

Compounds ^{a,b} / Cultivars ^{c}	CS	ML	CB	SH	SY	ZS-1	ZS-2	BP	MA	MI	SN	HS	ZH
Cinnamic acids (μg CAE/g DW)													
p-Coumaric acid	nd	nd	nd	nd	nd	nd	nd	nd	11.45 ± 0.17	3.70 ± 0.22	nd	9.85 ± 1.38	nd
Ferulic acid	nd	nd	nd	nd	58.44 ± 2.62	nd	19.97 ± 0.06	54.78 ± 3.05	nd	5.02 ± 2.35	nd	nd	17.30 ± 0.18
HE of caffeic acid	nd	nd	nd	nd	nd	nd	nd	nd	nd	nd	nd	nd	nd
HE of p-coumaric acid	nd	nd	nd	nd	nd	nd	nd	nd	nd	nd	16.36 ± 1.85	nd	nd
HE of ferulic acid	nd	tr	nd	nd	nd	nd	0.81 ± 0.35	131.33 ± 24.04	10.29 ± 0.38	4.06 ± 3.01	6.31 ± 0.77	10.26 ± 2.15	14.38 ± 1.46
Fertaric acid	nd	nd	85.17 ± 3.42	60.37 ± 3.38	nd	29.92 ± 0.61	33.08 ± 0.82	nd	118.19 ± 1.87	196.63 ± 8.67	nd	25.11 ± 4.88	10.49 ± 2.63
Total ^{d}	1.33 ± 0.00 ^{a}	tr	109.73 ± 2.51 ^{g}	70.53 ± 2.93 ^{e,f,g}	78.80 ± 2.57 ^{f,g}	38.08 ± 0.46 ^{a,b,c,d,e}	61.90 ± 0.97 ^{d,e,f}	216.19 ± 27.17 ^{i}	174.13 ± 1.29 ^{h}	229.37 ± 7.66 ^{i}	22.68 ± 1.07 ^{a,b,c,d}	55.04 ± 0.49 ^{c,d,e,f}	49.83 ± 1.18 ^{b,c,d,e,f}
Benzoic acids (μg GAE/g DW)													
HE of protocatechuic acid	0.91 ± 0.08	nd	6.65 ± 0.93	8.66 ± 0.57	6.36 ± 0.10	3.10 ± 1.54	7.26 ± 1.11	5.72 ± 1.43	7.09 ± 0.11	5.24 ± 0.37	12.59 ± 0.81	nd	3.02 ± 1.72
protocatechuic acid	nd	nd	nd	nd	1.56 ± 0.14	nd	nd	nd	nd	nd	nd	nd	nd
p-Hydroxybenzoic acid	nd	nd	nd	nd	nd	nd	nd	3.70 ± 0.03	nd	3.40 ± 0.04	nd	nd	nd
Ethyl gallate	nd	nd	nd	nd	nd	nd	nd	nd	nd	13.05 ± 0.26	nd	nd	nd
HE of vanillic acid	7.73 ± 0.38	10.17 ± 1.95	2.84 ± 0.18	6.64 ± 0.58	nd	3.43 ± 0.17	4.28 ± 2.04	3.45 ± 3.33	nd	nd	15.27 ± 2.09	7.32 ± 3.25	11.17 ± 2.26
Total ^{d}	8.64 ± 0.30 ^{b,c,d,e,f}	10.17 ± 1.95 ^{b,c,d,e,f,g}	9.49 ± 0.74 ^{b,c,d,e,f,g}	15.30 ± 0.01 ^{f,g,h,i,j}	7.92 ± 0.03 ^{b,c,d,e}	6.53 ± 1.38 ^{a,b,c}	11.54 ± 0.93 ^{c,d,e,f,g}	12.86 ± 1.93 ^{c,d,e,f,g}	7.09 ± 0.11 ^{a,b,c}	21.70 ± 0.60 ^{j,k,l}	27.85 ± 1.28 ^{l}	7.32 ± 3.25 ^{a,b,c,d}	14.19 ± 0.54 ^{d,e,f,g,h}

Appendix 2. *Cont.*

Compounds [a,b] \ Cultivars [c]	CS	ML	CB	SH	SY	ZS-1	ZS-2	BP	MA	MI	SN	HS	ZH
Ellagic acids (μg EAE/g DW)													
Ellagic acid-rha	nd	nd	nd	nd	nd	nd	nd	nd	nd	nd	nd	nd	nd
HHDP-galloylglucose	nd	nd	nd	nd	nd	nd	nd	nd	nd	nd	nd	nd	nd
HHDP-glucose	nd	nd	nd	nd	nd	nd	nd	nd	nd	nd	nd	nd	nd
Ellagitannin 1	nd	nd	nd	nd	nd	nd	nd	nd	nd	nd	nd	nd	nd
Ellagitannin 2	nd	nd	nd	nd	nd	nd	nd	nd	nd	nd	nd	nd	nd
Total [d]	nd	nd	nd	nd	nd	nd	nd	nd	nd	nd	nd	nd	nd
Stilbenes (μg RE/g DW)													
trans-Piceid	tr	5.01 ± 0.71	nd	nd	tr	tr	nd	nd	nd	8.67 ± 3.16	nd	3.64 ± 0.05	3.64 ± 0.05
trans-Resveratrol	26.83 ± 0.86	162.05 ± 6.49	nd	3.79 ± 0.45	nd	nd	2.83 ± 0.37	22.37 ± 0.80	2.17 ± 0.14	67.09 ± 2.44	27.31 ± 5.15	tr	tr
Total [d]	26.83 ± 0.86 [c]	167.06 ± 7.21 [e]	tr	nd	3.79 ± 0.45 [a]	tr	tr	2.83 ± 0.37 [a]	22.37 ± 0.80 [bc]	2.17 ± 0.14 [a]	75.76 ± 0.72 [d]	27.31 ± 5.15 [c]	3.64 ± 0.05 [a]

Compounds [ab] \ Cultivars [c]	ZY	BS	CT	CC	NR	MF	CH	SC	AL	NB
Flavonols (μg QE/g DW)										
Dk-3-glc	nd	nd	nd	nd	nd	nd	nd	nd	nd	133.35 ± 11.36
K-3-hex	nd	nd	47.07 ± 12.57	29.68 ± 1.68	53.11 ± 0.78	nd	nd	nd	nd	nd
Q-3-hex	nd	nd	21.36 ± 0.24	18.10 ± 0.59	28.70 ± 0.71	nd	16.05 ± 1.82	nd	43.20 ± 4.08	18.40 ± 2.46
M-3-gcn	nd	nd	nd	nd	nd	nd	nd	nd	nd	nd
Dq-3-hex	nd	nd	12.49 ± 5.64	nd	12.80 ± 2.71	nd	nd	8.87 ± 0.47	nd	nd
M-3-gal	nd	11.34 ± 2.65	nd	nd	nd	nd	nd	nd	nd	nd
M-3-glc	47.20 ± 1.42	12.52 ± 2.79	nd	19.58 ± 0.95	nd	nd	20.50 ± 3.01	18.94 ± 2.53	30.12 ± 15.98	38.50 ± 17.82
M-3-rha	nd	nd	nd	nd	37.72 ± 2.05	nd	nd	nd	238.31 ± 23.13	193.38 ± 40.61

Appendix 2. *Cont.*

Cultivars[c] / Compounds[ab]	ZY	BS	CT	CC	NR	MF	CH	SC	AL	NB
Flavonols (μg QE/g DW)										
Q	34.02 ± 1.58	95.29 ± 2.18	nd	nd	nd	69.21 ± 5.41	39.19 ± 0.65	nd	557.43 ± 44.70	452.60 ± 60.33
Dq-3-rha	nd	nd	9.41 ± 0.07	69.93 ± 3.29	nd	nd	nd	nd	nd	nd
Q-3-gal	19.49 ± 2.15	16.40 ± 1.09	nd	nd	nd	nd	19.46 ± 0.94	nd	nd	nd
Q-3-gcn	54.81 ± 3.16	192.28 ± 6.81	88.97 ± 2.60	122.82 ± 5.75	212.12 ± 5.05	35.54 ± 5.02	272.54 ± 0.89	55.90 ± 2.76	nd	nd
Q-3-glc	85.48 ± 5.37	131.29 ± 8.46	68.01 ± 0.36	111.25 ± 4.98	369.49 ± 17.69	7.71 ± 0.66	166.18 ± 0.78	35.54 ± 4.27	nd	nd
Q-3-rut	nd	19.51 ± 4.48	nd	278.92 ± 35.91	nd	nd	18.03 ± 0.15	100.95 ± 4.11	nd	nd
Q-3-rha	nd	30.65 ± 0.20	nd	57.43 ± 6.93	176.20 ± 15.77	nd	15.13 ± 1.97	nd	630.48 ± 61.26	675.03 ± 77.85
L-3-glc	nd	13.21 ± 3.69	nd	nd	nd	nd	12.96 ± 0.67	11.26 ± 1.98	nd	nd
K-3-gal	nd	nd	nd	nd	nd	nd	nd	nd	nd	26.66 ± 18.68
Q-3-xyl	nd	33.98 ± 4.14	nd	nd	nd	26.25 ± 1.32	16.25 ± 6.23	nd	240.55 ± 39.02	247.53 ± 0.17
I-3-xyl	nd	nd	nd	nd	40.20 ± 1.25	nd	nd	nd	nd	28.30 ± 26.95
K-3-rha	nd	nd	nd	nd	nd	nd	nd	nd	64.65 ± 4.70	63.09 ± 2.39
Dk-3-rha	nd	nd	nd	17.70 ± 2.59	nd	nd	nd	nd	nd	nd
K-3-glc	nd	nd	nd	nd	nd	nd	nd	nd	26.09 ± 2.43	15.69 ± 1.27
L-3-acglc	nd	nd	nd	nd	nd	nd	nd	nd	nd	nd
I-3-glc	nd	nd	12.07 ± 0.45	nd	33.31 ± 2.94	nd	nd	nd	nd	nd
I-3-rha	nd	nd	nd	nd	nd	nd	nd	nd	26.16 ± 7.08	nd
S-3-glc	nd	27.28 ± 1.74	17.72 ± 3.58	nd	nd	nd	15.97 ± 0.85	nd	nd	nd
Dq-3-acglc	nd	23.57 ± 3.37	nd	nd	nd	nd	nd	44.81 ± 2.69	nd	nd

Appendix 2. *Cont.*

Compounds[ab] \ Cultivars[c]	ZY	BS	CT	CC	NR	MF	CH	SC	AL	NB
Flavonols (μg QE/g DW)										
K-3-rut	nd	19.51 ± 4.48	nd	475.01 ± 22.17	61.20 ± 0.52	nd	nd	100.54 ± 2.66	nd	nd
Total [d]	241.00 ± 13.68 [a,b,c,d,e]	626.81 ± 25.98 [h,i]	277.09 ± 13.61 [a,b,c,d,e]	1200.41 ± 34.15 [j,k]	1024.86 ± 47.31 [j]	138.70 ± 12.40 [a,b,c]	612.25 ± 6.95 [g,h,i]	376.81 ± 10.77 [d,e,f]	1856.99 ± 202.38 [l]	1892.53 ± 53.60 [l]
Flavan-3-ols (μg CE/g DW)										
Gallocatechin	nd	nd	nd	nd	nd	nd	nd	nd	15.34 ± 0.21	20.72 ± 0.80
Epigallocatechin	nd	nd	nd	nd	nd	nd	nd	nd	21.05 ± 2.07	33.45 ± 3.21
Catechin	nd	nd	nd	25.68 ± 0.09	nd	15.85 ± 0.34	25.90 ± 5.36	nd	65.71 ± 2.95	nd
Epicatechin	nd	nd	nd	nd	nd	nd	nd	nd	163.53 ± 16.15	284.03 ± 34.18
Procyanidin dimer 1	nd	6.45 ± 0.64	9.66 ± 1.64	155.58 ± 3.39	1198.00 ± 25.56	nd	154.99 ± 10.24	55.80 ± 14.96	31.55 ± 3.06	113.28 ± 10.79
Procyanidin dimer 2	nd	nd	nd	nd	nd	nd	nd	nd	53.44 ± 12.22	nd
Procyanidin dimer 3	nd	nd	nd	nd	nd	nd	nd	nd	nd	nd
Procyanidin trimer	nd	nd	nd	nd	45.67 ± 2.98	nd	nd	nd	nd	nd
Total [d]	nd	6.45 ± 0.64 [a]	9.66 ± 1.64 [a]	181.26 ± 3.30 [a,b,c]	1243.67 ± 28.54 [f]	15.85 ± 0.34 [a]	180.89 ± 15.60 [a,b,c]	55.80 ± 14.96 [a]	350.62 ± 36.67 [b,c,d,e]	451.48 ± 48.98 [d,e]
Cinnamic acids (μg CAE/g DW)										
Chlorgenic acid	tr	7.66 ± 0.16	0.38 ± 0.02	8.85 ± 0.09	7.64 ± 0.42	0.71 ± 0.32	7.09 ± 0.02	6.80 ± 0.00	7.89 ± 0.11	8.26 ± 0.12
Caffeic acid	nd	nd	0.95 ± 0.07	5.55 ± 0.01	nd	nd	nd	nd	nd	nd
Caftaric acid	nd	nd	nd	nd	nd	nd	nd	nd	nd	nd
p-Coumaric acid	nd	nd	nd	8.99 ± 0.10	nd	nd	23.75 ± 3.13	nd	14.80 ± 2.35	nd
Ferulic acid	36.13 ± 0.94	32.58 ± 1.90	17.02 ± 3.17	nd	nd	7.87 ± 1.07	nd	7.53 ± 5.54	nd	nd
HE of caffeic acid	nd	nd	nd	nd	nd	nd	nd	nd	35.29 ± 4.58	nd
HE of p-coumaric acid	nd	nd	nd	nd	nd	nd	nd	9.79 ± 2.45	nd	8.29 ± 0.72
HE of ferulic acid	183.29 ± 39.52	9.19 ± 0.81	10.36 ± 0.34	26.55 ± 1.46	6.64 ± 1.02	1.25 ± 0.03	2.23 ± 0.34	8.97 ± 3.34	nd	8.34 ± 1.68

Appendix 2. *Cont.*

Cultivars[c] / Compounds[ab]	ZY	BS	CT	CC	NR	MF	CH	SC	AL	NB
Cinnamic acids (μg CAE/g DW)										
Fertaric acid	nd	nd	nd	27.86 ± 1.32	6.15 ± 0.44	3.47 ± 0.58	18.15 ± 0.10	nd	nd	nd
Total [d]	219.42 ± 38.59 [i]	49.43 ± 2.86 [b,c,d,e,f]	28.72 ± 2.88 [a,b,c,d]	77.80 ± 0.13 [f,g]	20.43 ± 1.01 [a,b,c]	13.29 ± 0.14 [a,b]	51.22 ± 3.59 [b,c,d,e,f]	33.08 ± 6.43 [a,b,c,d,e]	57.98 ± 6.81 [c,d,e,f]	24.89 ± 2.53 [a,b,c,d]
Benzoic acids (μg GAE/g DW)										
HE of protocatechuic acid	11.51 ± 0.22	0.19 ± 0.02	tr	nd	nd	nd	8.08 ± 0.03	5.05 ± 0.35	7.07 ± 0.73	11.34 ± 1.47
protocatechuic acid	nd	nd	nd	nd	nd	nd	nd	nd	nd	nd
p-Hydroxybenzoic acid	nd	nd	nd	nd	nd	nd	nd	nd	nd	nd
Ethyl gallate	nd	3.78 ± 1.20	nd	nd	nd	6.08 ± 0.01	nd	4.57 ± 2.16	nd	nd
HE of vanillic acid	4.39 ± 0.57	10.28 ± 2.19	0.58 ± 0.33	12.73 ± 0.14	3.43 ± 0.92	tr	6.30 ± 0.10	4.80 ± 2.54	6.96 ± 0.18	9.75 ± 2.08
Total [d]	15.90 ± 0.35 [g,h,i,j]	14.25 ± 3.41 [e,f,g,h,i]	0.58 ± 0.33 [a]	12.73 ± 0.14 [c,d,e,f,g]	3.43 ± 0.92 [a,b]	6.08 ± 0.01 [a,b,c]	14.39 ± 0.13 [e,f,g,h,i]	14.42 ± 4.35 [e,f,g,h,i]	14.03 ± 0.91 [d,e,f,g,h]	21.09 ± 3.56 [i,j,k,l]
Ellagic acids (μg EAE/g DW)										
Ellagic acid-rha	nd	nd	nd	nd	nd	nd	nd	nd	32.97 ± 9.04	nd
HHDP-galloylglucose	nd	nd	nd	nd	nd	nd	nd	nd	95.42 ± 3.21	184.91 ± 16.89
HHDP-glucose	nd	nd	nd	nd	nd	nd	nd	nd	nd	10.82 ± 1.47
Ellagitannin 1	nd	nd	nd	nd	nd	nd	nd	nd	93.45 ± 33.69	92.37 ± 35.32
Ellagitannin 2	nd	nd	nd	nd	nd	nd	nd	nd	303.32 ± 86.53	359.59 ± 98.08
Total [d]	nd	nd	nd	nd	nd	nd	nd	nd	525.16 ± 123.42 [a]	647.68 ± 148.81 [b]
Stilbenes (μg RE/g DW)										
trans-Piceid	nd	nd	nd	nd	nd	nd	nd	nd	tr	56.41 ± 8.18
trans-Resveratrol	tr	nd	nd	nd	nd	tr	tr	nd	12.15 ± 4.17	15.26 ± 4.33
Total [b]	nd	tr	nd	nd	nd	tr	tr	nd	12.15 ± 4.17 [a,b]	71.67 ± 12.51 [d]

nd means not detected. tr means trace. Q, quercetin; K, kaempferol; Ir, isorhamnetin; L, laricitrin; S, syringetin; Dq, dihydroquercetin; Dk, dihydrokaempferol; gal, galactoside; gcn, glucuronide; rha, rhamnoside; caglc, (6-caffeoyl)-glucoside; hex, hexoside; xyl, xyloside; rut, rutinoside. HE, hexose ester; rha, rhamnoside; [a] Numbers of each phenolic group represents non-anthocyanin phenolic compounds, see appendix 4; [b] Values are means of duplicate determination ± S.D.; [c] Each abbreviation represents a cultivar, see Table 1; [d] Different letters in each column are significantly different at 0.05 level from ANOVA.

© 2012 by the authors; licensee MDPI, Basel, Switzerland. This article is an open access article distributed under the terms and conditions of the Creative Commons Attribution license (http://creativecommons.org/licenses/by/3.0/).
